# A radiometric timescale challenges the chronology of the iconic 1992 Guliya ice core

**DOI:** 10.1126/sciadv.adx8837

**Published:** 2025-09-12

**Authors:** Shugui Hou, Theo M. Jenk, Wei Jiang, Wangbin Zhang, Huanting Hu, Xin Feng, Hao Li, Shuang-Ye Wu, Hongxi Pang, Jinhai Yu, Renhui Huang, Zheng-Tian Lu, Guo-Min Yang, Michael Bender, Margit Schwikowski

**Affiliations:** ^1^ School of Oceanography, Shanghai Jiao Tong University, Shanghai 200240, China.; ^2^ School of Geography and Ocean Science, Nanjing University, Nanjing 210023, China.; ^3^ PSI Center for Energy and Environmental Sciences, CH-5232 Villigen PSI, Switzerland.; ^4^ Oeschger Centre for Climate Change Research, University of Bern, Sidlerstrasse 5, CH-3012 Bern, Switzerland.; ^5^ Hefei National Research Center for Physical Sciences at the Microscale, School of Physical Sciences, University of Science and Technology of China, Hefei 230026, China.; ^6^ Hefei National Laboratory, University of Science and Technology of China, Hefei 230088, China.; ^7^ Department of Geology and Environmental Geosciences, University of Dayton, Dayton, OH 45469, USA.; ^8^ Department of Geosciences, Princeton University, Princeton, NJ 08544, USA.

## Abstract

The Guliya (Tibet) ice core drilled in 1992 (GP1992) has garnered special interest because of its exceptionally long timescale of ~760 thousand years. This timescale makes GP1992 currently the second oldest ice core in the world, much older than any other extrapolar ice cores. Here, we revisit the GP1992 timescale by dating a Guliya ice core (GP2021) that was drilled close to the GP1992 drilling site in 2021. All our data, including the absolute dates deduced from ^210^Pb, ^39^Ar, and ^14^C, confirmed an age of <3 thousand years at 175.1 meters depth of GP2021, compared with ~100 thousand years previously estimated at the stratigraphically equivalent depth in GP1992. Our results resolved several discrepancies between the GP1992 and other paleoclimate records in the region, leading to different insights in climate history of the Tibetan Plateau.

## Teaser

Radiometric timescale of a Guliya ice core drilled in 2021 challenges the chronology of the 1992 Guliya ice core.

## INTRODUCTION

**Fig. 1.**
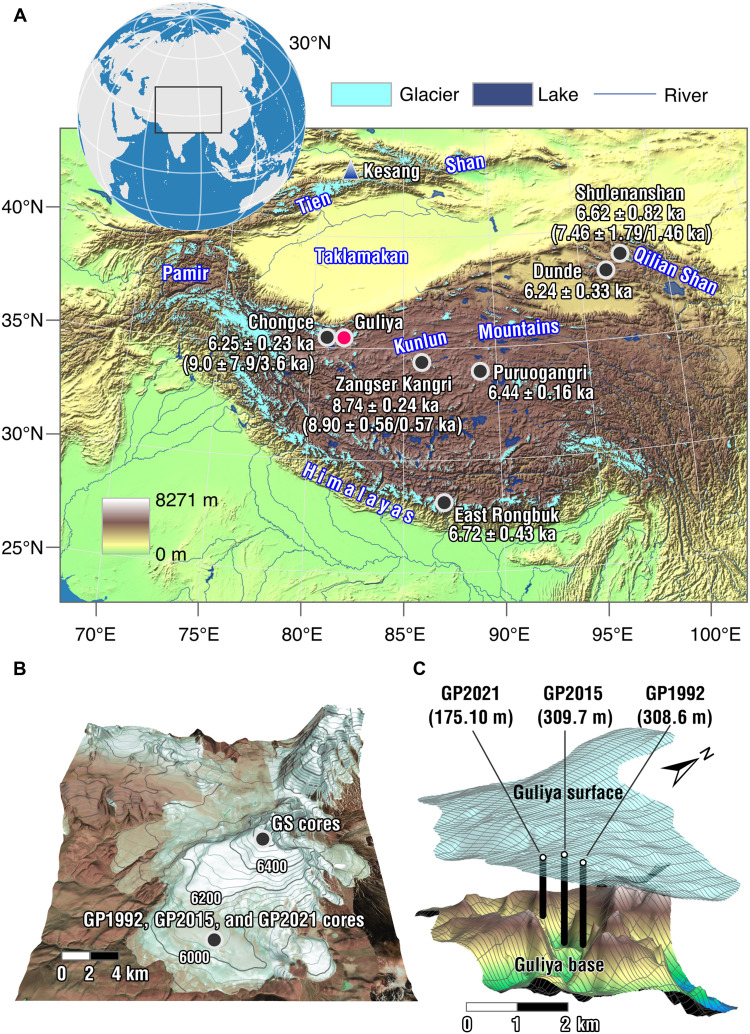
Ice core drilling locations on the Tibetan Plateau. (**A**) Topographic map showing the Tibetan ice core drilling sites. The numbers below each drilling site except Guliya indicate the oldest ^14^C calibrated ages for each corresponding ice core, and the numbers inside the brackets below the Chongce, Zangser Kangri, and Shulenanshan ice cores are the bottom ice ages estimated by a two-parameter glacier flow model constrained by absolute ^14^C calibrated ages (*20*). Glacier data are from the Global Land Ice Measurements from Space (available at http://glims.org, last access: 4 August 2020) (*86*). The topographic data are extracted from ETOPO1 elevation global data, available from National Oceanic and Atmospheric Administration (NOAA) at http://ngdc.noaa.gov/mgg/global/global.html (last access: 4 August 2020) (*87*). (**B**) A three-dimensional (3D) satellite map showing the locations of the Guliya ice core drilling sites. The GP1992, GP2015, and GP2021 drilling sites are too close to be distinguished and are shown as a single dot. The drilling sites (6710 m above sea level) of the three GS cores are also shown as a single dot. (**C**) The schematic depicts ice thicknesses and locations of the GP1992, GP2015, and GP2021 drilling sites. Ice thickness data are from (*74*) deposited in the NOAA National Climate Data Center. The bars in [(B) and (C)] represent lateral scales based on vertical projection.

The Guliya ice core (GP1992, 308.6 m in length) was drilled to bedrock in 1992 through the Plateau of the Guliya ice cap in the northwestern Tibetan Plateau, at 35°14′N, 81°28′E (Fig. 1 and fig. S1). Thompson *et al.* (*1*) established a timescale for GP1992 through a number of methods. They dated the ice at the bottom based on the assumption that the decreasing ^36^Cl concentration of ice at the very bottom of the core was primarily due to radioactive decay, leading to an age estimate of 760 ka (thousand years) at the bed (*1*). This basal age is far older than all other extrapolar ice cores. It would make GP1992 the second oldest continuous ice core in the world, only after the European Project for Ice Coring in Antarctica (EPICA) ice cores at Dome C, Antarctica (*2*). Thompson *et al.* (*1*) also wiggle-matched variations in the oxygen isotopic composition (δ^18^O_ice_; see Materials and Methods for definition) of GP1992 to those of the paleoatmospheric CH_4_ concentrations and δ^18^O_ice_ in the Greenland GISP2 ice core (*3*). On the basis of this matching, they derived a stratigraphically continuous climate record of the past 110 ka for the top 266 m of GP1992 (*1*).

In 2015, another ice core drilled to bedrock (GP2015, 309.7 m in length) was retrieved close to the GP1992 drilling site (Fig. 1C and fig. S1). In addition, three ice cores to bedrock were retrieved at the Summit of the Guliya ice cap (hereafter referred as GS2015, 50.72, 51.38, and 50.86 m in length, respectively) (Fig. 1B). Thompson *et al.* (*4*) established a chronology of the 50.86-m GS2015 core by matching its paleoatmospheric isotopic composition of O_2_ (δ^18^O_atm_) preserved in air bubbles with the well-dated δ^18^O_atm_ record of the West Antarctic ice sheet (WAIS) ice core (*5*). They then transferred this GS2015 chronology to GP2015 by matching their δ^18^O_ice_ profiles (*4*), resulting in an age of 15 ka at 144.4 m depth in GP2015. Furthermore, Thompson *et al.* (*6*) inferred an age of 41 ka at 187.4 m of GP2015 based on a ^36^Cl peak, which they attributed to the Laschamp Geomagnetic Event. They also argued that a ^36^Cl peak at 179 m depth could, given their timescale, be associated with the Mono Lake event, a possible geomagnetic event dated to 30 to 36 ka (*6*). Although a timescale below 187.4 m of GP2015 has not been published, the high consistency between timescales proposed for GP2015 and GP1992, at least until the inferred age of 41 ka, was invoked to support the accuracy of the Guliya Plateau (GP) ice core chronology at millennial to precessional timescales (*6*). In addition, Thompson *et al.* (*7*) counted annual layers using the seasonal variations of δ^18^O_ice_ and Cl^−^, Na^+^, and Ca^2+^ concentrations in the upper part of the GP ice cores. This work gave a continuous timescale back to 1840 CE. It should be noted that no absolute dating was conducted in any of the GP1992 and GP2015 timescale studies.

On the basis of the timescale outlined above, GP1992 would provide a continuous climate record of the last glacial stage (*1*), playing an important role for studying orbital variations in the Tibetan climatic record (*1*, *6*). This record was also punctuated by a sequence of stadial and interstadial events, as well as abrupt climate changes, such as ~100 oscillations with an average period of 200 years between 15 and 33 ka (*1*). It has since been widely cited by numerous studies [e.g., (*8*–*13*)]. The PAGES2k initiative used the GP1992 δ^18^O_ice_ record as a regional temperature proxy (*13*).

The timescales of GP1992 and GP2015 are largely based on the specific features of their ^36^Cl profiles (*1*, *6*). However, there are concerns with their interpretations. For example, the ^36^Cl excursion of GP2015 that was attributed to the Laschamps event is not a unique feature of the record (*6*). Thompson *et al.* (*6*) also showed other ^36^Cl excursions at shallower depths (fig. S2), for which they provided no explanations. The sampling resolution for ^36^Cl was increased around the depth assumed for the Laschamps event (fig. S2), facilitating the “discovery” of a geomagnetic excursion, whereas the other depths were discontinuously sampled. With such a sampling strategy, other potential ^36^Cl excursions would not be detected.

In addition, the ^36^Cl in the bottom ice of GP1992 could only provide a reliable age constraint if no processes other than radioactive decay cause ^36^Cl to decrease. The concentration of Cl falls by about 90% in the bottom few meters of the GP1992 core (fig. S3 and table S1). This result suggests Cl^−^ loss in the bottom ice of GP1992, which should also remove ^36^Cl chemically, without extensive radioactive decay of ^36^Cl. Instability of ^36^Cl deposits was observed in central East Antarctica and attributed to sublimation of H^36^Cl during snow metamorphism and ventilation of the firn layers (*14*). The measured ^36^Cl deposition flux of GP1992 was clearly lower than that at other mid-latitude glaciers and was also remarkably lower than the modeled ^36^Cl deposition flux (*15*). All of these suggest that ^36^Cl is not a reliable tool for dating ice cores from low accumulation areas, such as the Guliya ice cap (*14*).

Other studies proposed a much younger age constraints for the GP ice cores. Tian *et al.* (*16*) used ^81^Kr to radiometrically date the discharging ice sampled at the outskirts of the Guliya ice cap and obtained an upper age limit of the Guliya ice in the range of 15 to 74 ka. The large uncertainty is a consequence of using an isotope (^81^Kr) with a long half-life (2.29 × 10^5^ years) to date young samples. The GP1992 chronology is also put in doubt by the timescales of other Tibetan ice cores (*17*–*19*). For instance, the basal ages of two ice cores drilled to bedrock from the Chongce ice cap, ~30 km from the Guliya ice cap (Fig. 1A), were estimated to be $8.3\pm_{3.6}^{6.2}$ and $9.0\pm_{3.6}^{7.9}$ ka, respectively (*17*). Hou *et al.* (*18*) further suggested that the GP1992 and Chongce ice cores might cover a similar temporal span because of the significant similarity of their relative depth profiles of δ^18^O_ice_ (fig. S4). In addition, all other Tibetan ice cores, except Guliya, have been dated within the Holocene (Fig. 1A) (*20*).

To help resolve questions around the GP1992 and GP2015 timescales, we retrieved a Guliya ice core in 2021 (GP2021) close to the GP1992 and GP2015 drilling sites (Fig. 1C and fig. S1). GP2021 did not reach bedrock due to unexpected field difficulties and has a length of 175.1 m. We applied multiple independent radiometric and isotopic methods to establish the depth-age relationship of GP2021. We used absolute dating methods based on ^210^Pb, ^39^Ar, and ^14^C. We identified the 1963 CE nuclear bomb test horizon based on ^3^H activity. The appropriate age ranges with the respective radionuclides vary according to their half-lives, with ~0.15 ka for ^210^Pb (*21*, *22*), ~0.1 to ~1.8 ka for ^39^Ar (*23*), and ~0.5 to ~50 ka for ^14^C (*24*, *25*). ^39^Ar dating using atom trap trace analysis (ATTA), a laser-based single atom counting method (*26*), became only recently available for dating ice core samples with a precision better than ±20% (*23*). This bridged the age gap between ^210^Pb and ^14^C.

All of these radiometric methods (^210^Pb, ^39^Ar, and ^14^C) have previously been successfully applied for dating ice cores, including those from the Tibetan Plateau (*17*, *19*, *22*, *23*). In this study, these three radiometric methods were combined to date an ice core. We further analyzed the isotopic composition of trapped atmospheric O_2_ (δ^18^O_atm_) as another independent dating approach (*4*, *27*) and the isotopic composition of the ice (δ^18^O_ice_) for comparison with GP1992 and GP2015. Additional details about the drilling site, sample processing, and analytical methods are provided in Materials and Methods and the Supplementary Materials (figs. S6 to S13 and tables S2 to S7).

## RESULTS

### Depth-age relationship of GP2021

**Fig. 2.**
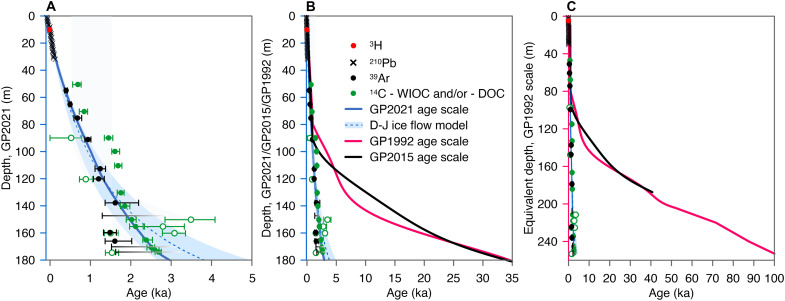
The depth-age relationships of the GP ice cores. (**A**) The depth-age relationship of GP2021. (**B**) The depth-age relationships of GP2021, GP2015, and GP1992 based on the currently established timescale for GP2021 and the original timescales for GP2015 (*4*, *6*) and GP1992 (*1*, *63*). (**C**) The stratigraphically equivalent depth-age relationships of GP2021, GP2015, and GP1992 referenced on the depth scale of GP1992 determined by matching their δ^18^O_ice_ profiles as shown in Fig. 4. The modeled depth-age relationship of GP2021 [D-J model; (*28*)] was constrained by the absolute ages deduced from ^210^Pb, ^39^Ar, ^14^C, and the ^3^H horizon of 1963 CE (dashed blue line, with shaded 1σ confidence band). The thick line indicates the GP2021 timescale, for which variability in annual net accumulation rates was allowed in the model (±15%). ^14^C results are either from the WIOC or DOC fraction or a statistical combination of both. Open symbols are statistical outliers in the ^14^C dataset (out of sequence). ^39^Ar ages at the depths of ~147, ~170, and ~174 m display large uncertainties due to contamination correction. For those, only their lower age limits could be established (horizontal black fading bars). The depth-age relationship of GP1992 is from (*1*), complemented with results from (*63*) for its top 80-m section.

We used the absolute ages obtained from ^210^Pb, ^39^Ar, and ^14^C analyses and the ^3^H horizon corresponding to 1963 CE to establish the depth-age relationship of GP2021 (Fig. 2; also see Materials and Methods). The resulting depth-age relationship was examined by the Dansgaard-Johnsen model [D-J model; (*28*)]. The ice becomes older with increasing depth, reaching $2.70_{-0.24}^{+1.76}$ ka at the bottom of GP2021 (175.1 m). The model simulations show that no exceptionally strong thinning or a marked change in accumulation rates are needed to be consistent with the observed ages from ^14^C and ^39^Ar dating (Fig. 2). Moderate thinning is expected for GP2021 layers given its depth of 175.1 m, about 55 m above bedrock (fig. S10).

The depth-age relationships of GP2021, GP1992 (*1*), and GP2015 (*4*, *6*) are comparable for their upper layers down to a depth of 80 to 90 m (Fig. 2B). Below that depth, they start to diverge greatly, which can only be explained by exceptionally rapid layer thinning or an extreme change in accumulation rates at the drilling sites, beginning at ~80 m depth for GP1992 and ~90 m depth for GP2015 (Fig. 2B). Such thinning was neither confirmed by the absolute ages from ^39^Ar or ^14^C dating in GP2021, nor could it be simulated by the ice flow model (Fig. 2A). If the comparison was made based on stratigraphically equivalent depth of the three GP ice cores, based on the excellent match of their δ^18^O_ice_ profiles (see the “Matching the δ^18^O_ice_ profiles of the GP ice cores” section below), the age divergence between GP2021 and GP1992/GP2015 becomes even more remarkable, up to an order of magnitude or more for their overlapping depths (Fig. 2C). Since the GP2021 timescale is based on two completely independent and absolute dating methods (i.e., ^14^C and ^39^Ar), and the two methods agree well within the scope of their uncertainties, this divergence suggests serious errors in the timescales of GP1992 and GP2015.

### Constraint of the GP2021 timescale from the isotopic composition of atmospheric O_2_ (δ^18^O_atm_)

**Fig. 3.**
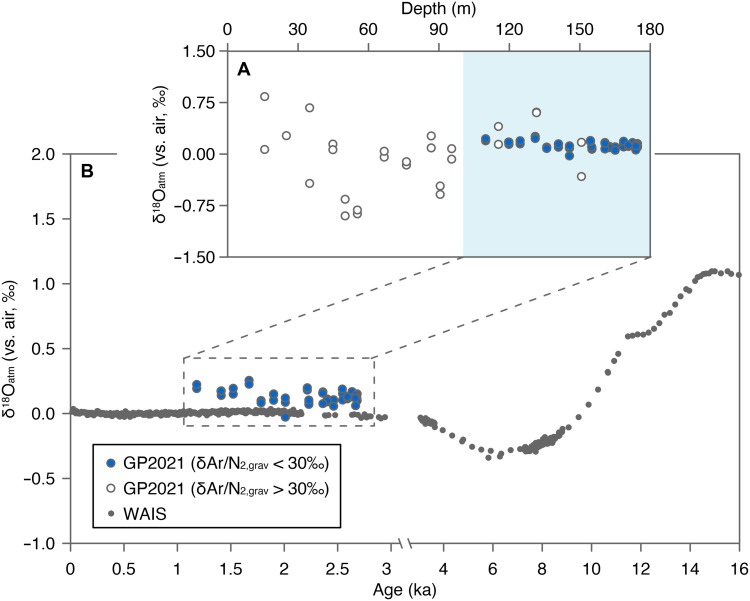
The δ^18^_atm_ profiles of the GP2021 and Antarctic WAIS ice cores. (**A**) The δ^18^O_atm_ profile versus depth of GP2021. (**B**) Selected δ^18^O_atm_ values least affected by melting, as inferred by δAr/N_2,grav_ ratios of <30 ‰, plotted on the WAIS δ^18^O_atm_ record (*5*). Note that the scale of *x* axis varies before and after 3 ka.

O_2_ is a well-mixed gas in the atmosphere, given that its residence time (>1000 years) is much longer than the mixing time of the atmosphere (~1 year). The long-term variation of its isotopic composition (δ^18^O_atm_) has been used to correlate ice cores or establish the depth-age relationship of undated ice core by matching its δ^18^O_atm_ stratigraphy to the well-documented global record (*29*). This dating approach was recently applied to the 50.86-m GS2015 ice core by Thompson *et al.* (*4*) and the Chongce ice core by Hu *et al.* (*27*). Figure 3 shows the δ^18^O_atm_ depth profile of GP2021, plotted against the δ^18^O_atm_ record of the WAIS ice core (*5*). The GP2021 data are much noisier than the WAIS record, especially above 100 m depth. The larger scatter is common for extrapolar ice core δ^18^O_atm_ records, because these cores are generally recovered from warmer areas where microbial respiration, melting, and other postdepositional processes can alter δ^18^O_atm_ (*4*, *27*). To minimize some of these effects, we first identified samples affected by melting based on δAr/N_2,grav_ values of trapped gases (see Materials and Methods for definition). We removed all samples with δAr/N_2,grav_ values higher than +30 per mil (‰) (*27*), which corresponds to an anomalous Ar enrichment of about 3% (fig. S13). For the remaining samples, the δ^18^O_atm_ values (mean value = 0.13 ± 0.05‰, *N* = 46 including duplicate or triplicate analysis of samples) are slightly above the atmospheric value of the past ~3 ka (~0‰), as recorded in the WAIS ice core (*5*). This is likely a consequence of microbial respiration, which has been previously observed in the upper sections of Tibetan ice cores (*4*, *27*). Respiration consumes the heavy ^18^O more slowly than ^16^O, thereby elevating δ^18^O_atm_ of residual O_2_ (see more details in Materials and Methods). In the WAIS record, δ^18^O_atm_ decreases between 3 and 6 ka (*5*), but no trend was observed in the GP2021 δ^18^O_atm_ data (Fig. 3). In particular, the high δ^18^O_atm_ values (up to 1.1‰) of GS2015 characteristic of the late glacial glacial (*4*) were not observed for GP2021. Thus, GP2021 should cover a period younger than 3 ka. This independent constraint is entirely compatible with the depth-age scale of GP2021 as shown in Fig. 2A and incompatible with the depth-age scales of GP1992 and GP2015.

### Matching the δ^18^O_ice_ profiles of the GP ice cores

**Fig. 4.**
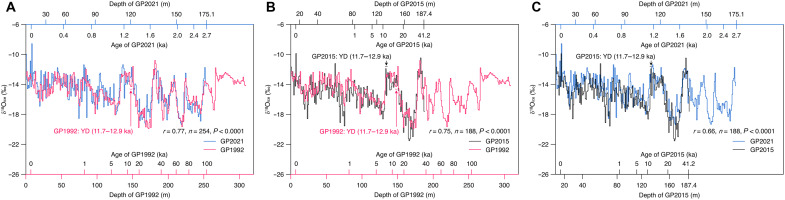
The δ^18^_ice_ profiles of GP2021 (blue lines), GP1992 (red lines), and GP2015 (black lines). (**A**) Matching the δ^18^O_ice_ profiles between GP2021 and GP1992. (**B**) Between GP2015 and GP1992. (**C**) Between GP2021 and GP2015. The Match software (*30*) was used for matching the δ^18^O_ice_ depth profiles of the GP cores. The timescales were also plotted along the depth axis according to the established depth-age relationship of each GP core. The timescale of GP2021 is according to the depth-age relationship presented in Fig. 2A. The timescales of GP1992 and GP2015, with the most distinctive signal previously interpreted as the YD event, are from (*1*, *4*), respectively. The δ^18^O_ice_ profile of GP1992 was digitalized from (*84*). The δ^18^O_ice_ profile of GP2015 was from (*6*), publicly available for the top 187.4 m only.

We compared the δ^18^O_ice_ profiles of GP2021 and GP1992 (Fig. 4A), GP2015 and GP1992 (Fig. 4B), and GP2021 and GP2015 (Fig. 4C) based on the Match software (*30*). This software uses dynamic programming to find the optimal alignment of two stratigraphic datasets through penalty functions. It produces objective and high-resolution results without automated correlation techniques or hand tuning (*30*). On the basis of the Match result, the 175.1-m GP2021 δ^18^O_ice_ record correlates with that of the GP1992 core to ~253 m depth (Fig. 4A), while GP2015 at 187.4 m depth corresponds to ~191 m depth in GP1992 core (Fig. 4B). We obtained a positive significant correlation between GP2021 and GP1992 (*r* = 0.77, *n* = 254, *P* < 0.0001) and a slightly weaker but significant correlation between GP2015 and GP1992 (*r* = 0.75, *n* = 188, *P* < 0.0001) and between GP2021 and GP2015 (*r* = 0.66, *n* = 188, *P* < 0.0001) (Fig. 4). The significant similarities of the three GP δ^18^O_ice_ profiles imply high reproducibility of the GP δ^18^O_ice_ records. This allows us to transfer depth of one core to another based on the stratigraphic equivalency. The timescales of GP2015 and GP1992 are generally consistent, with GP2015 being slightly older than GP1992 at their stratigraphically equivalent depth (Fig. 4B). However, the timescale of GP2021 is markedly different from that of GP1992 and GP2015 (Figs. 2, B and C, and 4, A and C). For example, the bottom depth of 175.1 m (corresponding to $2.70_{-0.24}^{+1.76}$ ka) for GP2021 is stratigraphically equivalent to the depth of ~253 m for GP1992, where the ice age of GP1992 is ~100 ka according to its original timescale (*1*). To further examine the age divergence of the three GP ice cores, we compiled a list of original ages of GP1992 at selected depths (*1*) and compare them with the corresponding ages at each stratigraphically equivalent depth of GP2015 (*4*, *6*) and GP2021 (Fig. 4 and Table 1). It is apparent that the ages of GP1992 and GP2015 start to get much older below 80 m depth (Table 1). The discrepancies increase markedly with depth (Fig. 2, B and C).

**Table 1.** Comparison of ages at selected stratigraphically equivalent depths of GP1992, GP2015, and GP2021. The stratigraphically equivalent depths are determined from their matching profiles of δ^18^O_ice_ versus depth, shown in Fig. 4. Ages of GP1992 marked with an asterisk are from (*7*). Ages for other samples of GP1992 are from (*1*). Ages of GP2015 are from (*6*) and ages of GP2021 from this work (Fig. 2A).

Depth of GP1992 (m)	Age of GP1992 (ka B.P.)	Equivalent depth of GP2015 (m)	Age of GP2015 (ka B.P.)	Equivalent depth of GP2021 (m)	Age of GP2021 (ka B.P.)
20	~0.07*	27.0	0.07	22.6	$0.07_{-0.01}^{+0.04}$
40	~0.24*	41.6	0.14	42.6	$0.24_{-0.06}^{+0.05}$
60	~0.45*	53.6	0.19	64.6	$0.50_{-0.14}^{+0.05}$
80	~0.76*	72.2	0.53	78.4	$0.68_{-0.19}^{+0.06}$
100	3.25	97.2	2.01	91.4	$0.88_{-0.25}^{+0.08}$
120	5.70	119.2	5.81	103.2	$1.06_{-0.28}^{+0.14}$
140	8.20	139.6	13.56	114.2	$1.22_{-0.28}^{+0.24}$
144	10.40	144.4	15	117.4	$1.27_{-0.29}^{+0.27}$
160	19.60	160.2	20.86	127.8	$1.46_{-0.30}^{+0.38}$
180	34.40	182.0	35.44	138.4	$1.70_{-0.33}^{+0.52}$
187	38.30	187.4	41	141.0	$1.76_{-0.33}^{+0.56}$
200	46.00	–	–	144.4	$1.83_{-0.33}^{+0.63}$
220	71.60	–	–	156.8	$2.15_{-0.32}^{+0.94}$
240	87.60	–	–	168.0	$2.46_{-0.27}^{+1.39}$
253	~100.00	–	–	175.1	$2.70_{-0.24}^{+1.76}$

## DISCUSSION

The timescale of GP2021 is established through multiple independent measures, including the absolute ages deduced from ^210^Pb, ^39^Ar, and ^14^C measurements. Our results cast serious doubts on the validity of the original GP1992 and GP2015 timescales (*1*, *4*, *6*). Consequently, many conclusions drawn from these timescales need to be revisited. For example, on the basis of their original timescales, the δ^18^O_ice_ records of GP1992 and GP2015 show high values during the Younger Dryas (YD) event (12.9 to 11.7 ka). This result is unexpected because the YD event was a cold interval in the Northern Hemisphere (*31*), whereas modern δ^18^O in precipitation indicated positive relationship between δ^18^O in precipitation and temperature on the northern Tibetan Plateau (TP) (*32*). Thompson *et al.* (*6*) proposed an inverse relationship between δ^18^O_ice_ and temperature during the YD event, as well as during Heinrich event 1 (H1), due to a glacial/postglacial isotopic switch, resulting in δ^18^O_ice_ enrichment during the cold YD event and H1 event. However, this interpretation is not supported by a transient simulation of climate-isotope coevolution over the last deglaciation (20 to 11 ka), which shows modest δ^18^O_ice_ depletion during the H1 and YD events (*33*). This problem can be resolved by our different understanding of the timescales of the GP ice cores. On the basis of the matching of GP2021 and GP1992 (Fig. 4A), the depth previously assigned to YD event for GP1992 (*1*) should correspond to ~1.31 to 1.34 ka, an interval within the cold Late Antique Little Ice Age (*34*, *35*).

In addition, we examined the previously claimed ~100 δ^18^O_ice_ oscillations with an average period of 200 years between 15 and 33 ka, deduced from the GP1992 record (*1*). We chose the high-resolution δ^18^O_ice_ profile of GP1992 from (*36*, *37*) for a better comparison with that of GP2021. On the basis of their stratigraphic equivalency, 18 to 37 ka in the GP1992 δ^18^O_ice_ profile corresponds to 1.44 to 1.77 ka in the GP2021 δ^18^O_ice_ profile (fig. S14), a period of climate shift from the Roman Warm Period to the Dark Age Cold Period (*35*). We further identified periods of 2 to 5 years for the GP2021 δ^18^O record during this interval (fig. S15). This is obviously different from the previously suggested average period of 200 years based on the original chronology of GP1992 (*1*).

The timescale for GP2021 gives different insights about the regional climate of the past three millennia. The increasing GP2021 δ^18^O_ice_ during the Common Era (fig. S16A) suggests a millennial warming trend given the observed positive relationship between δ^18^O in precipitation and temperature on the northern TP (*32*). Given our current understanding on the δ^18^O_ice_ record of the GP cores, the increasing trend of the GP2021 δ^18^O_ice_ record during the Common Era (fig. S16A) might imply a millennial cooling trend instead. This conclusion is consistent to the PAGES2k reconstructions (*13*, *38*). In addition, the GP2021 δ^18^O_ice_ record shows significant multidecadal to multicentennial periods (fig. S17) and large variations (up to ~8‰) at multidecadal to multicentennial scales before ~800 CE (fig. S16C). Positive δ^18^O_ice_ excursions occurred roughly at 1.2 to 1.4, 1.7, 1.9, 2.2, and 2.6 ka (fig. S16C). The absolutely dated calcite δ^18^O record of Kesang stalagmites also shows large fluctuations at multidecadal to multicentennial scales superimposed on an increasing δ^18^O trend over the past 3 ka (*39*). The δ^18^O_ice_ record of GP2021 is significantly and positively correlated with the Kesang stalagmite δ^18^O record (*r* = 0.462, *P* < 0.001, *n* = 90) (fig. S18). This is reasonable because the Kesang stalagmite δ^18^O variations largely reflect changes in the δ^18^O of precipitation (*39*). The Kesang record supposedly shows possible incursions of Indian Summer Monsoon (ISM) rainfall and related moisture during past wet periods (*39*), while changes of moisture source (*40*) and moisture pathways (*41*) also play an important role in controlling the Tibetan ice core δ^18^O_ice_ variations. In particular, the Guliya ice cap is located in the transition zone between ISM and the mid-latitude westerlies (*42*). Both paleoclimate data and model studies have revealed strong variability in the strength of the Atlantic Meridional Overturning Circulation at multidecadal to centennial scales (*43*, *44*), which substantially shifts the position of westerlies and the intertropical convergence zone and hence ISM by altering the temperature gradient between low and high latitudes (*45*). As a result, the Guliya δ^18^O_ice_ may reflect the net results of regional hydroclimate change in the northwestern TP connected with interplay between ISM and westerlies (*46*), rather than solely temperature variations (*1*, *6*). It is worth pointing out that this study focused primarily on the chronologies of the GP ice cores. Future work will be conducted on the detailed forces and mechanism of past climate variations on the Tibetan Plateau. We further suggest that measurements of δ^18^O_atm_, ^39^Ar, ^14^C, ^81^Kr, and possibly ^41^Ca for the deeper sections of the GP1992, GP2015, and, hopefully, new GP cores to the bottom will provide a complete and robust timescale for the Guliya ice cores.

## MATERIALS AND METHODS

### The δ notation

Here, stable isotope ratios are reported in the δ notation defined as

$$\delta =\left[\frac{R_{\text{sample}}}{R_{\text{reference}}}-1\right]\times 1000$$ (1)

where R indicates the ratio between the heavier and lighter isotope providing the per mil (‰) deviation from a reference standard.

Specifically, the oxygen isotopic compositions of ice are expressed in δ notations (‰) as

δOice18=(Rsample18RVSMOW18−1)×1000 (2)

where ^18^R is the isotopic ratio of ^18^O/^16^O for ice, R_sample_ is the isotopic ratio of a sample, and R_VSMOW_ is the ratio of Vienna standard mean ocean water (VSMOW).

Similarly, for ice core trapped gases of N_2_, O_2_, and Ar, the isotopic compositions and gas ratios are defined as

δN15=(Rsample15Rair15−1)×1000 (3)

δO(O2)18=(Rsample18Rair18−1)×1000 (4)

$$\delta O_{2}/N_{2}=\left(\frac{O_{2}/N_{2,\text{sample}}}{O_{2}/N_{2,\text{air}}}-1\right)\times 1000$$ (5)

$$\mathrm{\delta Ar}/N_{2}=\left(\frac{\text{Ar}/N_{2,\text{sample}}}{\text{Ar}/N_{2,\text{air}}}-1\right)\times 1000$$ (6)

where ^15^R is the isotopic ratio of ^15^N/^14^N for trapped N_2_, ^18^R is the isotopic ratio of ^18^O/^16^O for trapped O_2_, and O_2_/N_2_ and Ar/N_2_ are gas ratios. R_sample_ is the isotopic ratio of a sample, and R_air_ is the ratio of the well-mixed modern atmospheric ratio.

Because of the gravitational effects in the firn column, isotopic and gas ratios should be corrected for gravitational fractionations according to the following equation (*47*, *48*)

δOatm18=δO(O2)18−2·δ15N (7)

$$\delta O_{2}/N_{2,\text{grav}}=\delta O_{2}/N_{2}-4\cdot \delta^{15}N$$ (8)

$$\mathrm{\delta Ar}/N_{2,\text{grav}}=\mathrm{\delta Ar}/N_{2}-12\cdot \delta^{15}N$$ (9)

The coefficients for δ^15^N in Eqs. 7 to 9 are the differences in molecular weight between heavy and light gases in atomic mass units.

### The GP1992, GP2015, and GP2021 ice cores

The Guliya ice cap, situated on the south slope of the west Kunlun Mountains in the northwest Tibetan Plateau, is the largest continental-type (cold) ice cap in central Asia (Fig. 1 and fig. S1). The modern climate is predominantly governed by the interaction between the mid-latitude Westerlies and the Asian Summer Monsoon (*42*, *49*). On the basis of the monthly meteorological data extracted from the 10-km-resolution High Asia Refined reanalysis version 2 (*50*), the mean annual temperature at the GP is −14.1°C, and the mean annual total precipitation is ~642 mm, with ∼45% of precipitation falling in winter/spring (December to May or DJFMAM) and ∼55.5% in summer/autumn (June to November or JJASON; fig. S5).

The GP1992 ice core (308.6 m in length) was drilled in July/August 1992 (*1*) and the GP2015 core (309.7 m in length) in September/October 2015 (*4*, *6*). In May 2021, we revisited the Guliya ice cap and retrieved an ice core (GP2021, 175.10 m in length, 6061 m above sea level) at 35°13′48.91″N, 81°28′0.07″E on the GP, close to the GP1992 and GP2015 drilling sites (Fig. 1C and fig. S1). GP2021 did not reach bedrock. The measured temperature at 10 m depth of the GP2021 borehole was −13.4°C. GP2021 was retrieved with an electromechanical drill.

### Measurements of stable isotopes of ice core water samples (δ^18^O_ice_)

The GP2021 ice core was cut with a resolution of ~5 cm per sample, resulting in a total of 3515 samples. The water samples were analyzed for the composition of stable isotopes by a Wavelength Scanned Cavity Ring-Down Spectrometer (Picarro, USA, model: L2140-i) at Nanjing University. To reduce the influence of instrument drift in the water isotope measurements, internal water standard samples were inserted among the samples for measurements (one water standard sample for every seven samples). Each sample was measured eight times, with the first five measurements discarded to eliminate memory effects. The average of the last three measurements was calibrated on the basis of the linear regression between the known isotopic values of our three internal water standards and their measured values. The calibrated values of samples were taken as the test results. The stable isotopic ratio (δ^18^O_ice_) was calculated by Eq. 2. The reference is the VSMOW standard. The analytical precision is better than 0.1‰ (*51*).

### ^210^Pb dating

^210^Pb, a product of the natural ^238^U decay series, is suitable to date materials less than ~150 years old given its half-life of 22.3 years. For ^210^Pb dating of the GP2021 ice core, a total of 32 samples were collected continuously in 1-m resolution for depths between 0.5 and 32.5 m (table S2). The samples (between 186 and 193 g) were cut in a −20°C cold room at Nanjing University and then processed and analyzed at Paul Scherrer Institut (PSI), following the method established by Gäggeler *et al.* (*21*). Briefly, the ^210^Pb activity concentration was measured using α-counting of its granddaughter ^210^Po which reaches secular equilibrium with ^210^Pb of ∼1 ½ years after deposition. After 0.05% (v/v) of HCl (30%) and 100 μl of a ^209^Po standard with known activity were added to the samples, they were melted overnight. The solutions were then heated to 90°C for ∼10 hours. Reducing condition was established by bubbling SO_2_ gas through the liquid for 3 min. A silver disk of typically 6 mm in diameter was immersed into the liquid that was continuously mixed with a magnetic stirrer. To prevent Po deposition on the back side of the silver disk, it was mounted in a plastic frame. After drying, the disks were positioned in vacuum chambers at a distance of 1 mm from a silicon surface barrier detector (ORTEC, ruggedized, 300 and 450 mm^2^) having an α-energy resolution of ~23 keV full width at half maximum at 5.3 MeV. The ^209^Po standard was measured via its well separated 4.9-MeV α-line and lastly used to determine chemical yield of deposition (73 ± 11%). The overall yield including detection efficiency was 22 ± 4%. Counting times, dependent on the activity of the sample, were between 3 and 21 days. The ^210^Pb background (BGD) from supported lead was 1.5 ± 0.4 mBq kg^−1^.

The intercept of the linear regression of ln([^210^Pb activity]_i_), and *x*_i_, depth in meter water equivalent (m w.e.), yielded the ^210^Pb activity concentration at the surface of 185 ± 55 mBq kg^−1^ ([A_0_]). The corresponding slope (*s*) is 0.211 ± 0.009. Applying the law of radioactive decay under the assumption of constant initial deposition and constant accumulation, the final age–depth was obtained by Eq. 10, where *t* is the age in years since the date of drilling in 2021, *s* is the slope of the fit, *T*½ is the half-life of ^210^Pb (22.3 years), and *z* is the depth in m w.e. (table S2)

$$t=\frac{-s}{\text{ln}\left(2\right)}\times T_{1/2}\times z$$ (10)

Not provided in table S2 is the age of an individual samples (*t*_i_), which can be calculated from its ^210^Pb activity concentration ([A_i_])

$$t_{i}=\lambda^{-1}\left(\frac{A_{0}}{A_{i}}\right)$$ (11)

where λ is the decay constant of ^210^Pb (0.03114 year^−1^). The ^210^Pb activity concentration profile of the GP2021 core, as well as the depth-age relationship derived for the upper part of the core, is shown in fig. S6.

### Tritium horizon

Analysis of tritium (^3^H) was carried out for the GP2021 core on 20 samples collected continuously from a depth range between 8.4 and 12.4 m (corresponding to a sampling resolution of 0.2 m per sample). The selection of this depth interval was based on the prior ^210^Pb dating discussed above. Aliquots of 10 ml from the melted samples were analyzed at PSI using low-level scintillation counting (liquid scintillation counter: TriCarb 2770 SLL/BGO, Packard SA) with a total count time of 600 min in 3 cycles. The analytical limit of detection (LoD) was 1.5 Bq liter^−1^ [12.7 Tritium Units (TU)]. The activity concentration was less than the LoD for samples below 10.5 m depth. All ^3^H data presented in this work were decay-corrected to the reference year 1963 CE (i.e., corrected for the decay between 1963 and November 2021, the time of analysis). The profile of ^3^H activity concentrations in the GP2021 core is shown in fig. S7.

### ^39^Ar and ^85^Kr dating

For ^39^Ar and ^85^Kr dating, 12 samples were collected from different depths of the GP2021 core and analyzed (table S3). The outer layer of the ice was removed to avoid potential contamination from modern air. After this procedure, each ice sample weighed about 4 to 6 kg. The ice was melted inside a vacuumed container, and the released gas was then extracted and transferred into 50-ml stainless steel holders filled with activated charcoal (3 g). This method could achieve extraction efficiencies higher than 95% and contamination with modern air below 1‰ at a processing time of about 30 to 40 min per sample. The gas content for each sample was about 16 to 30 ml/kg, which is consistent with gas contents retrieved from other TP ice cores (*23*). Argon and krypton were separated from the extracted air with a purification system based on titanium gettering and gas chromatography (*52*), yielding krypton recoveries higher than 90%. From each ice sample, we obtained about 0.6 μl of standard temperature and pressure (STP) krypton.

The argon and krypton samples were analyzed separately with the ATTA method, a laser-based single atom counting method (*23*). The principle of ATTA and analysis procedure was reported in previous publications (*26*, *53*). ^39^Ar (or ^85^Kr) atoms were selectively captured by laser beams into a magneto-optical trap and counted through detecting their fluorescence. For each sample, the activity of ^85^Kr was denoted as R_85_, and the ^39^Ar abundance relative to modern argon of 2018 CE was denoted as R_39_. If a sample is more than 100 years old (age, >100 a) and no contamination is present, then R_85_ should be close to zero (R_85_ = 0). The age of the sample can then be calculated as follows

$$\text{Age}=-T_{1/2}\text{ln}\left(R_{39}\right)/\text{ln}2$$ (12)

where *T*_1/2_ is the half-life of ^39^Ar (*T*_1/2_ = 268 years).

If, however, ^85^Kr is found in samples older than 100 a, the samples are likely contaminated with modern air, and its ^39^Ar abundance must be corrected, using the following relationship

$$\begin{array}{c} R_{39\_\text{measure}}=R_{39\_\text{corrected}}\times \left(1-\eta \right)+R_{39\_\text{contamination}}\times \eta \end{array}$$ (13)

where η is the contamination fraction, and R_39_contamination_ is the ^39^Ar abundance in the contaminant. In our case, the dominant contamination is the modern air which enters the ice through postdepositional melting and refreezing, so R_39_contamination_ = 1. On the basis of this, the R_39_corrected_ can then be calculated as follows

$$R_{39\_\text{corrected}}=\left(R_{39\_\text{measure}}-\eta \right)/\left(1-\eta \right)$$ (14)

The contamination fraction η can be extracted using the measured ^85^Kr activity R_85_. Following the same relationship for ^39^Ar, we get the following for R_85_

$$\begin{array}{c} R_{85\_\text{measure}}=R_{85\_\text{corrected}}\times \left(1-\eta \right)+R_{85\_\text{contamination}}\times \eta \end{array}$$ (15)

where R_85_corrected_ = 0, since the ice sample is much more than 100 years old. In our case, the contaminant is modern air. We assumed an activity of ~80 ± 5 dpm/cc, i.e., R_85_contamination_ = R_85_modern air_. Therefore, the contamination fraction is derived as

$$\eta =R_{85\_\text{measure}}/R_{85\_\text{modern air}}$$ (16)

Contributions to the ^39^Ar uncertainties include:

1) Statistical errors for atom counting of ^39^Ar and ^85^Kr. Since the atom counts for both isotopes were low (10 to 100), we adopted the Feldman-Cousins method to estimate these errors (*54*). We used the measured ^85^Kr to estimate the effect of contamination on ^39^Ar and applied corrections accordingly as outlined above. The correction causes additional uncertainties which were included in the calculation.

2) Systematic error due to the uncertainty of the half-life of ^39^Ar (268 ± 8 years). This error causes the calculated ^39^Ar ages to increase or decrease for all ice samples.

3) A systematic age uncertainty of about ±20 years due to the uncertainty of the atmospheric ^39^Ar history (*55*).

Final ages were corrected assuming a Δ-age of 30 years [gas-ice age difference; (*23*)] toward older ages. The final overall uncertainties were then derived from the propagation of (i) the analytical uncertainty, (ii) the age uncertainty of ±3% due to the error of the ^39^Ar half-life, (iii) the systematic age uncertainty of about ±20 years due to the uncertainty of the atmospheric ^39^Ar history (*55*), and an uncertainty of ±20 years presumed for the correction of the Δ-age. In the end, ice core samples younger than 1.8 ka can now be dated with a precision better than ±20% with this method (*23*, *56*).

### ^14^C dating

#### Samples

For ^14^C analysis, samples were collected from the 2021 GP core at 16 discrete depth intervals. From each interval, two parallel samples were cut and prepared, one for ^14^C in the water-insoluble (WIOC) and the other for ^14^C dissolved (DOC) organic carbon fraction. The results are given in table S4.

#### Methods of ^14^C analysis

Samples were prepared and analyzed for ^14^C with an accelerator mass spectrometry (AMS) between December 2021 and February 2022. Samples for WIOC ^14^C dating were prepared following the protocol described by Uglietti *et al.* (*25*) with a summary outlined below. The samples were shipped frozen to PSI for processing and analysis. To eliminate potential contamination, we first removed ~3 mm of the outer layer of samples with a precleaned stainless steel bandsaw in a −20°C cold room and thoroughly rinsed them with ultrapure water. After melting the samples in closed, precleaned jars [1 liter, polyethylene terephthalate-glycol (PETG), Semadeni], we extracted the water-insoluble carbonaceous particles by filtering the samples through prebaked quartz fiber filters (Pallflex Tissuquartz 2500QAT-UP). The samples contained very high mineral dust content, similar to samples from the Chongce ice cores from the same region (*17*), but much higher than other TP ice cores. Therefore, two filters were used for each sample to prevent clogging (except for GA100, GA133, and GA234). Photos of filters in fig. S8 show the high dust load. We adapted the method of dust removal from Fang *et al.* (*57*) to increase the efficiency. To remove mineral carbonates, sample filters were acidified three times with 0.5 μl of 1 M HCl for 15 min each time. All these initial steps were performed in a class 100 laminar flow box to ensure clean conditions. At the University of Bern (Laboratory for the Analysis of Radiocarbon with AMS, LARA laboratory), the WIOC samples were combusted with a temperature of 375°C (below the carbonate combustion temperature) in a thermo-optical organic carbon and elemental carbon (OC/EC) analyzer (Sunset Modeldoc4L, Sunset Laboratory Inc., USA). The CO_2_ produced from the WIOC was then measured online for ^14^C concentration with a 200-kV compact AMS [MIni radioCArbon DAting System (MICADAS)] equipped with a gas ion source and a gas interface system. More details on sample preparation procedures, analytical methods, instrumentation, and postprocessing of data can be found in previous studies (*24*, *25*, *57*–*59*).

Samples for DOC ^14^C were prepared in the cold room in the same way as those for WIOC. Afterward, samples were rinsed for further decontamination under helium atmosphere in a dedicated system for DOC extraction. Full details for the DOC method can be found in Fang *et al.* (*57*). Briefly, DOC was converted to CO_2_ in a catalyzed ultraviolet oxidation reaction. In a vacuum line, the evolving CO_2_ was then cryogenically purified and flame sealed in glass ampules for final AMS analysis. AMS analysis was done with the MICADAS at LARA, which is coupled to an ampule cracker setup via the same gas interface also connecting the OC/EC analyzer used for WIOC analysis.

#### Notations and data processing

All ^14^C results are expressed as fraction modern (F^14^C), which is defined as the ^14^C/^12^C ratio of the sample divided by the same ratio of the modern standard referenced to the year1950 CE [National Institute of Standards and Technology (NIST) standard oxalic acid II, SRM 4990C], both being normalized to −25‰ in δ^13^C to account for isotopic fractionation. Daily AMS calibration was performed using sets of modern (NIST oxalic acid II, SRM 4990C, F^14^C = 1:3407 ± 0:0005) and fossil standards (sodium acetate, Sigma-Aldrich, no. 71180, F^14^C = 0:0018 ± 0:0005). Table S4 provides both the AMS F^14^C raw data and postprocessed F^14^C values after corrections for procedural blank contribution, constant contamination in the AMS system (background), and cross-contamination between AMS samples. The overall procedural blanks were estimated using artificial ice blocks of frozen ultrapure water, treated like environmental ice samples. For WIOC, the values applied for correction of the procedural blank were 1.3 ± 0.6 μg C (*n* = 146) for mass carbon (m_c_) and 0.69 ± 0.15 (*n* = 92) for F^14^C, in agreement with previously reported values (*58*, *59*). If sampled onto two filters, m_c_ used for WIOC correction was 1.6 μg C, accounting for the additional contribution of empty, prebaked filters. For DOC, the respective values were 1.9 ± 1.6 μg C (*n* = 42), with a F^14^C of 0:68 ± 0:13 (*n* = 39). For the WIOC OC/EC analyzer AMS system, the constant contamination is 0.91 ± 0.18 μg C with F^14^C of 0.72 ± 0.11. For the ampule cracking AMS system, the respective values were 0.06 ± 0.18 μg C with a F^14^C of 0.50 ± 0.11. AMS cross-contaminations were 0.5 and 0.2% of the previous sample F^14^C for the WIOC and DOC setup, respectively. All DOC results were further corrected for the contribution from ^14^C in situ production (*57*). For the Guliya site latitude and elevation, an in situ production rate of 500 ^14^C atoms g_ice_^−1^ year^−1^ was applied (*60*–*62*). The average accumulation rate was estimated to be 0.2 ± 0.05 m w.e. year^−1^ based on the ^210^Pb dating (0.15 m w.e. year^−1^ on average but not accounting for layer thinning). For comparison, previous results for the Guliya plateau from stake and snow pit measurements range from 0.14 to 0.22 m w.e. year^−1^ and reconstructed values from the GP1992 ice core (for the period 1500–1992 CE) suggest rates of more than 0.3 m w.e. year^−1^ for some periods (*7*, *63*–*65*). The incorporation of in situ ^14^C by DOC was considered to be 18 ± 7% (*66*). All uncertainties provided above were fully propagated throughout data processing and reflected in final calibrated ^14^C ages.

#### ^14^C calibration

Final F^14^C values were then calibrated against the Northern Hemisphere radiocarbon calibration curve IntCal20 (*67*), extended to the present with the post–bomb atmospheric curve of Northern Hemisphere zone 3 from Hua *et al.* (*68*). Calibration was performed using the online program OxCal v4.4.4 (*69*). Straightforward calibration is all that is required if the objects dated have no other chronological or stratigraphic information associated with them (*70*). Using WIOC or DOC for ^14^C dating allows sampling at desired depth and resolution/spacing along the core. This is highly beneficial compared to traditional carbon dating, which is dependent on random findings (or not) of macrofossils—e.g., plant or insect fragments—at arbitrary depths. As the samples for WIOC/DOC ^14^C analysis from the GP2021 core were extracted from more than 50 m above bedrock (see below for estimated ice thickness at the GP2021 drill site) where folding is unlikely, particularly on a large and relatively flat plateau, we can assume an increase in age with depth for these samples. We therefore used this additional information for calibration, using the OxCal in-built Bayesian sequence model (*71*, *72*). The calibration results are presented in fig. S9 with numbers provided in table S4. The calibrated ^14^C ages are indicated as years before present (cal B.P.; with B.P. referring to the year 1950 CE). They represent the median age of the derived probability distribution function, with the 1σ range indicating uncertainty.

#### 1D ice flow model and the development of the GP2021 timescale

The absolute dates developed by previous methods were used to constrain a one-dimensional (1D) ice flow model (D-J model) described by Dansgaard and Johnsen (*28*) to produce a continuous timescale for GP2021. The D-J model assumes steady-state conditions, using three parameters to relate depth and time: the ice thickness *H*, the average annual net accumulation rate *b*, and the shear zone thickness *h*. The model uses an approximation of horizontal ice flow velocities to calculate the vertical strain rate (thinning of annual ice layers with depth). This calculation assumes that the flow velocity described by Glen’s law is constant from the glacier surface down to a given height above bedrock, from which point it is then linearly decreasing with depth to zero at the bed. This depth is denoted as the shear zone thickness *h* (other expressions such as “thickness of bottom shear layer” or “kink height” were also used in the literature).

Because of their mathematical simplicity, 1D ice flow model allows for relatively straightforward optimization of parameters to achieve a best fit to dated layers. A wealth of approaches and applications can be found in the literature. In this study, optimization of parameters was achieved by minimizing the misfit between the D-J modeled ages and the absolute ages of dated layers, using least sum of time-weighted squares of residuals (*73*) with a generalized reduced gradient algorithm (GPR) for nonlinear optimization (Excel Solver). This approach could also accommodate for slight deviation from the steady state assumed by the original model, allowing a variable accumulation rate (*b*). To test the robustness of the model, we performed a series of experiments using different input datasets and parameter settings. All input data are presented in table S5. A summary of all modeling experiments and their results are presented in table S6, with details provided in the following.

1) For *H*, we used preset values based on glaciological and dating independent information. The ice thickness of 230 m at the GP2021 drill site was estimated from contour mapping of the ice thicknesses obtained with ground penetrating radar measurements (GPR) published in (*74*) with data available from the National Oceanic and Atmospheric Administration (NOAA) Paleo data repository. The uncertainty of this estimate is likely in the order of about 30 to 40 m (fig. S10). To explore a large range of possibilities, we also considered the bedrock depths of the GP1992 (309.7 m) and GP2015 ice cores (308.6 m), applying a preset value of 310 m. For *h*, the theoretically possible maximal range is 0 < *h* ≤ *H*, thus the “free” setting was constrained accordingly. For *h* = 0, the D-J model yields the same solution as the Nye model (*75*), yielding younger ages at depth compared to a value of >0. For high-altitude glaciers, to our knowledge, only one study derived an estimate for *h*. Gabrielli *et al.* (*76*) found *h* ≈ 0.6*H to be a good solution for a high-altitude ice core drill site in the European Alps (Mt. Ortles). They showed that with this estimate, the resulting horizontal velocities from the D-J model closely agreed with measurements. Accordingly, as a dating value independent of *h*, it was preset to *h* = 0.6*H. On the basis of their observations, Gabrielli *et al.* (*76*) also proposed that a generally applicable value of *h* might be derived from the depth at the firn-ice transition, where density reaches a value of ~0.85 g cm^−3^. In agreement with the observed absence of an extended firn pack around the drill site in 2021, densities around this value are reached within the first meter of the GP2021 ice core. This would suggest *h* ≈ *H* to be a good estimate for this site, see (2) below. Using different datasets, summarized in tables S4 and S5 and discussed below in more detail, the parameter for the annual net accumulation rate *b* was then optimized, to get best solutions.

2) Different datasets were used for parameter optimization of either *b* only (see above), *h* and *b*, or all three parameters *H*, *h*, and *b*. The data were selected to allow investigating not only the absolute ages from the different dating methods in combination (full dataset) but also individually for the results from ^39^Ar and ^14^C dating (with the age from ^3^H analysis considered in all cases for sufficient model constrain at shallow depths). To take potential limitations of the ^39^Ar and ^14^C dating methods into account, two subsets of each were used for parameter optimization. On the basis of the half-life of ^39^Ar, dating is applicable for samples in the age range from around 0 to 1.8 ka B.P. (*23*). Therefore, this method is likely best suited for the samples down to around 120 m depth. ^14^C ages for that depth range might be questionable considering the possibility that bias from carbonate contribution cannot be entirely excluded despite the improved methodology. On the other hand, ^14^C ages give robust results for depths >120 m. In this interval, ^14^C of DOC and WIOC are in particularly good agreement (fig. S9). ^14^C ages are more accurate than ^39^Ar ages for depths >120 m because samples fall outside of the optimal ^39^Ar dating range.

#### Modeling results and GP2021 timescale

Results for the modeling experiments are summarized in table S6, and modeling results of the age-depth relationship are shown in Fig. 2A and fig. S11. We found that the model that allows modest variability of *b* produced a result that best fits all observed data, and it was then used as the final GP2021 timescale (Fig. 2A).

For *H*, the values derived from optimization to fit to the absolute ages were between 251 and 277 m, with one exception yielding 310 m (for the full ^14^C dataset). This is within or close to the upper ice thickness of 270 m estimated from the contour mapping of the available ice thickness data (fig. S10). Values for *h* yielded with *h* = *H* in all cases if *H* was not fixed to a preset value. This agrees well with the hypothesis for a good estimate of *h* being the depth of the firn-ice transition from (*76*) (accordingly, in the absence of firn at GP2021 at the surface, or equal to *H*, respectively). The values derived for *b*, in the range from 0.12 to 0.21 m w.e. year^−1^, agree well with the values of 0.14 to 0.23 m w.e. year^−1^ reported previously for the GP during the recent period (*7*, *63*–*65*, *77*). They are reported to cover the past 500 years (back to 1500 CE). While one does not show a trend for this period, with an average of ~0.25 m w.e. year^−1^ and variations between <0.1 and ~0.4 m w.e. year^−1^ (*63*), the reconstruction from *77* indicates a remarkable trend of decreasing net accumulation rates from ~0.5 m w.e. year^−1^ in 1500 CE to ~0.2 m w.e. year^−1^ in 1992 CE. In either case, these overall higher rates compared to our results should be reflected in younger ages at equivalent depths (Fig. 4), compared to the ages determined in this study. This is what is observed when comparing the GP2015 with the GP2021 timescale. For the youngest period of the cores (500 years), the discrepancy between the age scales is moderate (Fig. 2A), especially if compared to the very large discrepancy observed with increasing depth.

In any case, a steady-state model is unable to reflect changes in annual accumulation rates. Therefore, the final GP2021 timescale was derived allowing for modest variability of *b*, slightly improving the fit to the data. With the approach, described in the study of Fang *et al.* (*57*), we used a smoothed five-point running mean of *b*. This running mean, calculated for the individual dated horizons, was used for model input instead of constant/steady-state *b* (see also fig. S11). We obtain variations in the range of 0.14 to 0.19 m w.e. year^−1^ (±15%).

### δ^18^O of O_2_

#### Analytical method

A total of 73 ice core samples were collected from 30 discrete depths between 15.7 and 174.5 m of GP2021 and analyzed for δ^18^O, δ^15^N, δO_2_/N_2_, and δAr/N_2_ of trapped air (table S7). The samples were cut in the −20${}^{{}^{\circ}}$ C walk-in cold room at Shanghai Jiao Tong University, with ~20 to 40 g of ice per sample. Two or three parallel samples were cut for each depth to allow for duplicate or triplicate analyses. Two to 3 mm of surface ice was trimmed away to minimize potential contamination.

The isotopic and elemental ratios of ice core trapped air were measured using a melt equilibrium method (*78*, *79*). Each ice sample was placed in a flask immersed in a cold ethanol bath (−30${}^{{}^{\circ}}$C). The flask was evacuated for 10 min until the internal pressure dropped to ~10^−5^ mbar. The sample flask was then sealed, and the ice inside was melted at room temperature (22${}^{{}^{\circ}}$ C) for release of the trapped air. Afterward, the flask was shaken for 1 hour on a shaker table to equilibrate gases in melt water and headspace. After discarding the melt water, the sample flask was connected to a vacuum line. The bottom of the flask was immersed in a cold ethanol bath (−30${}^{{}^{\circ}}$ C) to freeze any remaining water. The air was then released and passed through two traps at liquid nitrogen temperature (−196${}^{{}^{\circ}}$ C) to remove water vapor and CO_2_. Last, the remaining gas was collected in a sample finger filled with silica-gel particles (Sigma-Aldrich, 6-14 mesh) and then heated and released into a dual-inlet isotope ratio mass spectrometer (Thermo Fisher Scientific Delta V Plus) for measurement of δ^15^N, δ^18^O, δO_2_/N_2_, and δAr/N_2_.

Air standards were analyzed concurrently with the samples. During the analysis period, 43 atmospheric air samples were collected in the laboratory courtyard. The observed long-term precisions (1σ) for air standards were ±0.037‰, ±0.043‰, ±0.2‰, and ± 0.2‰ for δ^15^N, δ^18^O, δO_2_/N_2_, and δAr/N_2,_ respectively. The measured isotopic and elemental ratios were first corrected for the differences in the O_2_/N_2_ ratios between the sample and reference gases (*80*, *81*) and then reported relative to the mean observed values of air standards (Eq. 1).

#### Data processing

Isotopic and elemental ratios were first corrected for gravitational fractionations according to Eqs. 7 to 9. To evaluate gas loss fractionations, we compared the pair differences in δ^18^O, δO_2_/N_2,grav_, and δAr/N_2,grav_ (i.e., the difference between the first measured sample and the second or third measured samples) for samples of the same depth. We observed a scattering of pair difference values from shallower samples and those with high δAr/N_2,frav_ but no obvious trend from either the Δδ^18^O-ΔδO_2_/N_2,grav_ or the Δδ^18^O-ΔδAr/N_2,grav_ plots (fig. S12). This is expected because the GP2021 ice core was analyzed shortly after drilling and had not yet experienced gas losses. Consequently, no corrections for gas loss were necessary for the trapped air data of the GP2021 ice core.

#### Depth profile of δ^18^O_atm_ and age constraint for GP2021

δ^18^O_atm_ values are scattered and noisy (fig. S13), especially in samples above 100 m depth with high δAr/N2. Below 100 m, the reproducibility of δ^18^O_atm_ is notably better, with δAr/N_2,grav_ values close to zero. For samples above 40 m, the scattered δ^18^O_atm_ values are likely caused by partial bubble close-off. For samples between 40 and 100 m, both δO_2_/N_2,grav_ and δAr/N_2,grav_ values are notably higher than zero, likely caused by seasonal melting of surface snow (*82*). Ar and O_2_ are approximately twice as soluble as N_2_ (*83*). When surface snow melts, δAr/N_2_ and δO_2_/N_2_ values of dissolved gases in the melt water become higher, approaching +1255 and 1046‰ (at 0°C), respectively, as the melt water becomes saturated with these gases. δAr/N_2,grav_ and δO_2_/N_2,grav_ values between 40 and 100 m are higher than 100‰, indicating that the ice core samples contained more than 10% refrozen melt water (melt layers). The δ^18^O_atm_ values of samples containing a high fraction of melt layers can be either positive or negative, as O_2_ could fractionate in either direction depending on the dominating process. Equilibrium fractionation has a factor of +0.7‰, and kinetic fractionation has a factor of −2.2‰. The melt respiration correction method proposed in (*4*) is not applicable here. Instead, we followed the method described in (*27*), simply discarding all samples with δAr/N_2,grav_ values higher than 30‰. This approach ensures that the remaining samples contain less than 2 to 3% of melt layer, substantially reducing the δ^18^O_atm_ uncertainties caused by melting.

For samples with δAr/N_2,grav_ values less than 30‰, we observed good agreement in δ^18^O_atm_ in replicate samples from the same depth (pooled SD = 0.025‰, *N* = 46). δ^18^O_atm_ values from 100 to 174.5 m show similar isotopic compositions (mean value = 0.13 ± 0.05‰) with no obvious trend, but δ^18^O_atm_ values are slightly higher than the atmospheric value of the past 3 ka [which is ~0; (*5*)]. This positive deviation is likely due to small amount of microbial respiration observed for Tibetan ice cores in previous studies (*27*).

For these samples, the mean δAr/N_2,grav_ value is +10.8‰, but the mean δO_2_/N_2,grav_ value is −1.8‰. As O_2_ and Ar have similar solubility, the lower δO_2_/N_2,grav_ values are likely caused by additional microbial respiration, which consumes O_2_ in both gas bubbles and melt layers. Assuming solubility equilibrium of O_2_ and Ar in melt layers, the amount of O_2_ consumed by respiration can be estimated as

$$\delta O_{2}/N_{2,\text{res}}=\delta O_{2}/N_{2,\text{grav}}-\frac{1046‰}{1255‰}\mathrm{\delta Ar}/N_{2,\text{grav}}$$ (17)

Then, the magnitude of the δ^18^O_atm_ increase caused by respiration could be estimated on the basis of the closed-system Rayleigh fractionation equation

δOincrease=(δO2/N2,res/103−1)α−118 (18)

where α is the oxygen isotopic fractionation factor for respiration. The mean δO_2_/N_2,res_ value for selected samples below 100 m is −10.8‰, corresponding to +0.1 to +0.2‰ increase in δ^18^O_atm_ values when using an α value of 0.99 or 0.981 (*5*, *82*). This calculation explains the slightly positive δ^18^O_atm_ values observed for GP2021.

After being corrected for all possible postdepositional alterations, the δ^18^O_atm_ values of GP2021 samples are close to zero with no obvious trend. By comparing them with the δ^18^O_atm_ record of the WAIS ice core (*5*), we concluded that the ice of the GP2021 core at 174.5 m is younger than 3 ka, consistent with the timescale from radiometric dating (Fig. 2A).

#### Depth profiles in δ^18^O_ice_ of the GP ice cores

A direct comparison of GP1992, GP2015, and GP2021 δ^18^O_ice_ profiles by depth is challenging due to issues related to sampling resolution and data availability. The sampling resolutions for δ^18^O_ice_ measurements are ~2.4 cm per sample for GP1992 (*1*), ~3.3 cm per sample for GP2015 (*6*), and ~5 cm per sample for GP2021. The raw δ^18^O_ice_ data for GP1992 and GP2015 are not publicly available. The GP1992 δ^18^O_ice_ data deposited in the online NOAA repository were averaged with resolutions of 10, 5, 3, 1, and 0.6 m per point at the depth ranges of 0 to 100, 100 to 150, 150 to 252, 252 to 308, and 308 to 308.6 m respectively. Therefore, we extracted GP1992 δ^18^O_ice_ values averaged at a resolution of 1 m per point from (*84*) using the WebPlotDigitizer software. The GP2015 δ^18^O_ice_ data were from (*6*), only available for the top187.4 m. This depth corresponds to an age of 41 ka in the GP1992 timescale. To facilitate the comparison, we averaged the δ^18^O_ice_ values of the GP2015 and GP2021 at a resolution of 1 m per point and matched them with the 1-m digitalized GP1992 δ^18^O_ice_ profile using the Match software (*30*), and the results are shown in Fig. 4.

#### The GP2021 δ^18^O_ice_ time series

The δ^18^O_ice_ values of GP2021 vary from −25.19 to −2.88‰, with an average value of −15.04‰. Given the GP2021 sampling resolution of ~5 cm per sample, this corresponds to a temporal coverage from a few months in the ice core upper sections to over two years in the deep sections. To perform the time series analysis, the uneven δ^18^O_ice_ series was linearly interpolated to annual resolution using MATLAB software, resulting in an annual time series of GP2021 δ^18^O_ice_ back to 748 BCE.

We applied the Hilbert-Huang transform ensemble empirical mode decomposition (EEMD) method (*85*) to separate different modes of variability for the annual GP2021 δ^18^O_ice_ time series. The EEMD decomposed the record into 12 instrinsic mode functions (IMFs) loaded on different temporal scales. The composite IMFs 10 and 11 explain 13.6% of the total variance in the GP2021 δ^18^O_ice_ time series and reflect the long-term variation of the δ^18^O_ice_ record (fig. S16A), indicating the long-term warming trend during the past two millennia. The composite IMFs 6 to 9 explain 34.2% of the total variance for the GP2021 δ^18^O_ice_ record and reflect the multidecadal to multicentennial δ^18^O_ice_ variations, showing large δ^18^O_ice_ fluctuations before ~800 CE (fig. S16C). Spectral and wavelet analyses were also applied to determine the periodicities and periodic stabilities. The results show dominant periodicities of around 7, 9, 15, 22, 71 to 78, 90 to 100, 120 to 137, 164 to 185, 227 to 294, and 345 to 833 years (fig. S17).

## Supplementary Materials

### The PDF file includes:

Figs. S1 to S18 Tables S1 to S3, S5 to S7 Legend for table S4 References
