# Supplementary material for: A radiometric timescale challenges the chronology of the iconic 1992 Guliya ice core
Source: Sci Adv. 2025 Sep 12;11(37):eadx8837. doi: 10.1126/sciadv.adx8837 (PMC12429011; doi:10.1126/sciadv.adx8837)
Supplement: Supplementary file 1 — Figs. S1 to S18 Tables S1 to S3, S5 to S7 Legend for table S4 References [file sciadv.adx8837_sm.pdf]

Science Advances
AAAS

Supplementary Materials for

A radiometric timescale challenges the chronology of the iconic 1992 Guliya ice core

Shugui Hou *et al.*

Corresponding author: Shugui Hou, shugui@nju.edu.cn

Sci. Adv. **11**, eadx8837 (2025)
DOI: 10.1126/sciadv.adx8837

The PDF file includes:

Figs. S1 to S18
Tables S1 to S3, S5 to S7
Legend for table S4
References

Other Supplementary Material for this manuscript includes the following:

Table S4

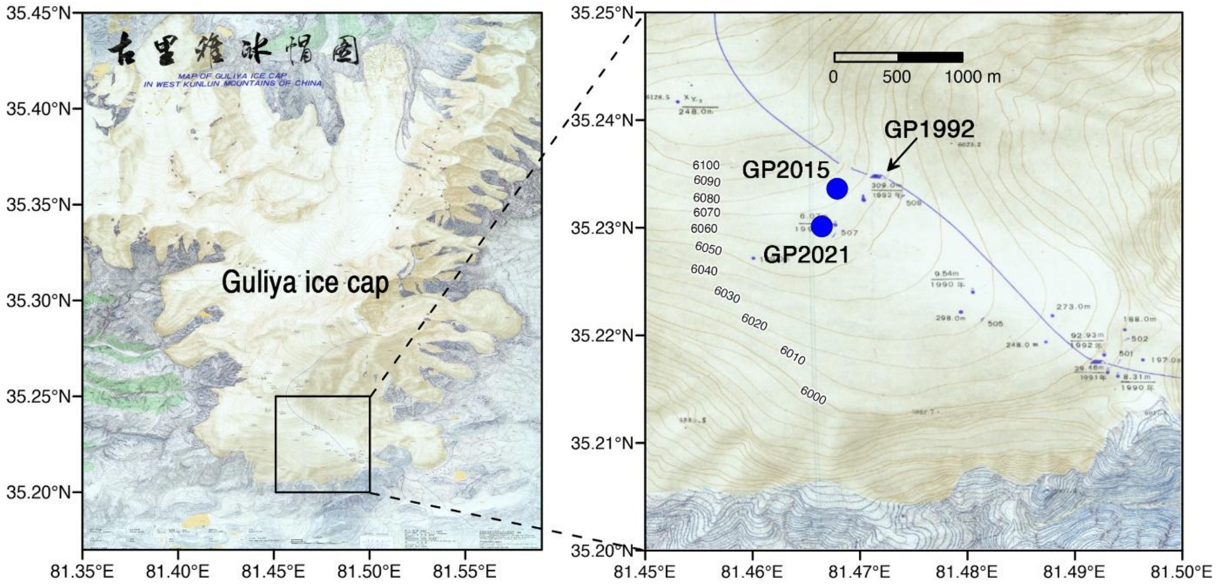

Fig. S1. Map of the Guliya ice cap in the western Kunlun Mountains. Maps adapted from ref. *88*. Accordingly, the elevation of the GP1992 drilling site is between 6060-6070 m above sea level, consistent with the elevation of the GP2021 drilling site at 6061 m above sea level, which is slightly lower than the elevation of 6200 m above sea level as reported in ref. *1*. The background map was reprinted with permission from Xi’an Cartographic Publishing House, 1992. All rights reserved.

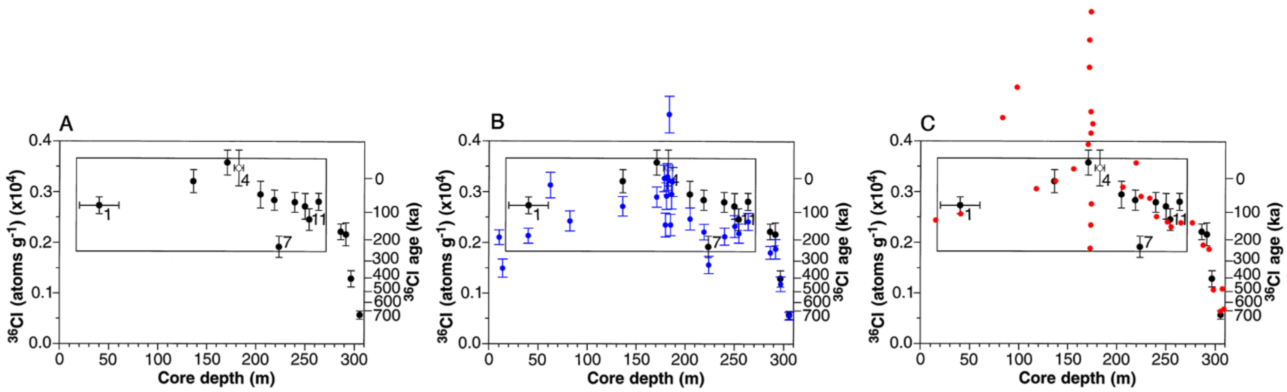

Fig. S2. The profiles of ^{36}Cl concentrations vs. ages for the ice cores of GP1992 and GP2015. (A) The ^{36}Cl profile of GP1992, shown as Figure 4A in ref. *1*. (B) The updated ^{36}Cl profile for GP1992 (blue dots) that were deposited at: https://www.ncei.noaa.gov/access/paleo-search/study/12426, overlaid on the original ^{36}Cl profile of GP1992 shown in (A). (C) The ^{36}Cl profile of GP2015 (red dots) that were digitalized from Figure 2 in ref. *6*, overlaid on the original ^{36}Cl profile of GP1992 shown in (A).

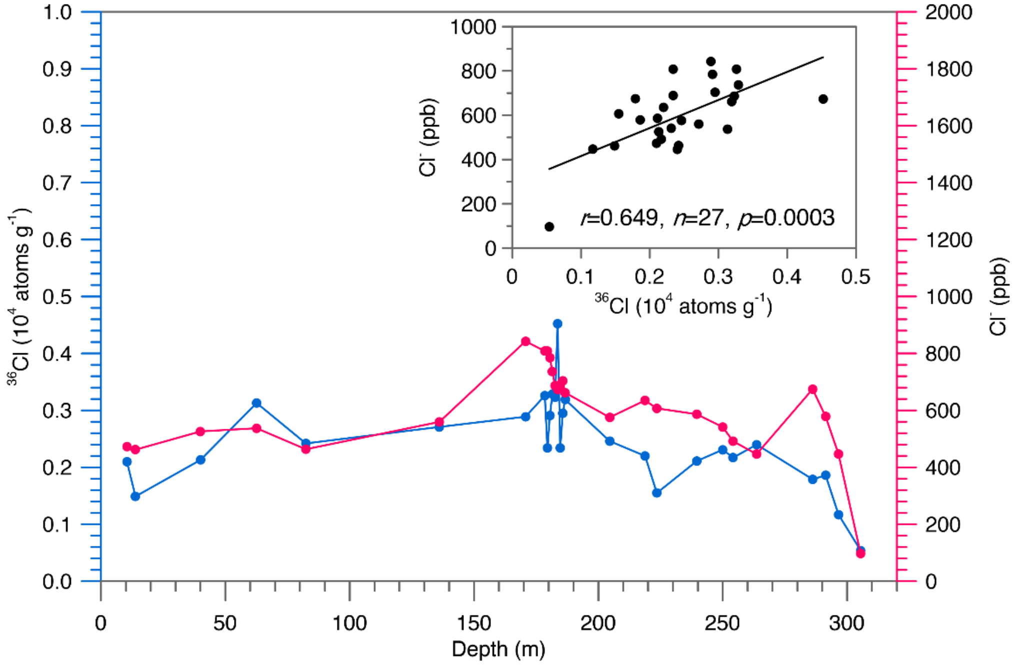

Fig. S3. The ^{36}Cl (blue line) and chloride (pink line) concentration profiles of GP1992. The inset shows the correlation between ^{36}Cl and chloride. The original GP1992 ^{36}Cl and chloride data were available at: https://www.ncei.noaa.gov/access/paleo-search/study/12426. A total of 27 samples were analyzed for ^{36}Cl concentrations. The chloride concentration data were averaged with resolutions of 10 m, 5 m, 3 m, 1 m and 0.6 m per point at the depth ranges of 0-100 m, 100-150 m, 150-252 m, 252-308 m and 308-308.6 m, respectively (*1*). For correlation analysis, the chloride concentration data at the depth of ^{36}Cl concentration samples were obtained using a simple linear interpolation (the interpolated data can be found in Table S1).

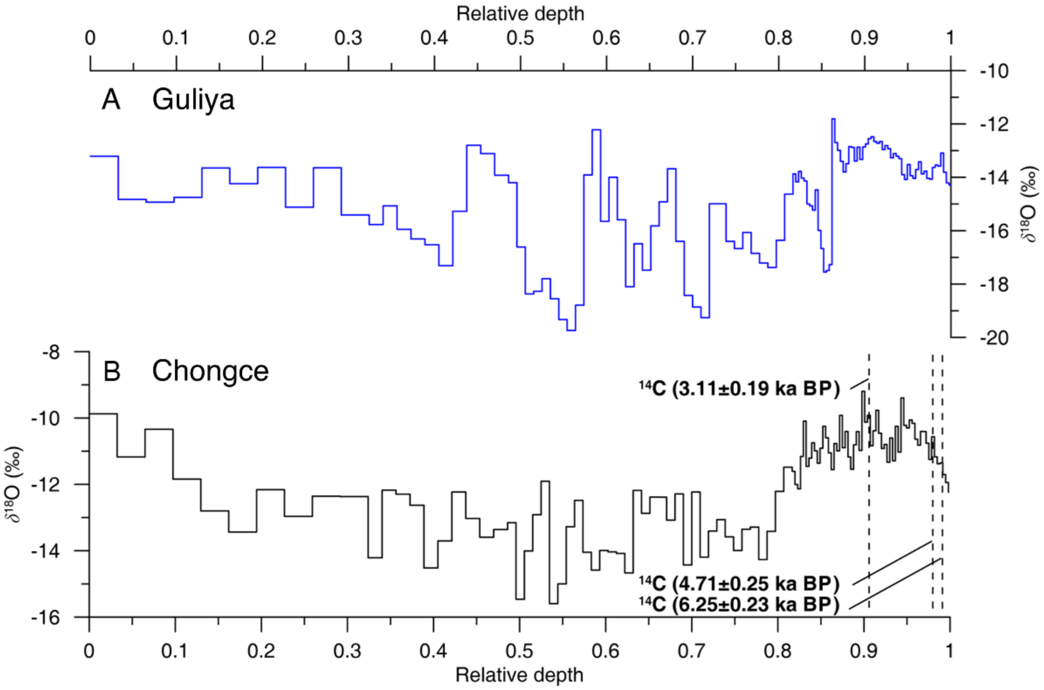

Fig. S4. The $\delta^{18}O_{ice}$ profiles of GP1992 (A) and Chongce (B) ice cores, plotted against their relative depth. A significant positive correlation is observed between the $\delta^{18}O_{ice}$ profiles of GP1992 and Chongce ice cores ($r=0.57$, $n = 110$, $p<0.001$). Modified from ref. *18*, the CC BY 4.0 license at https://creativecommons.org/licenses/by/4.0/

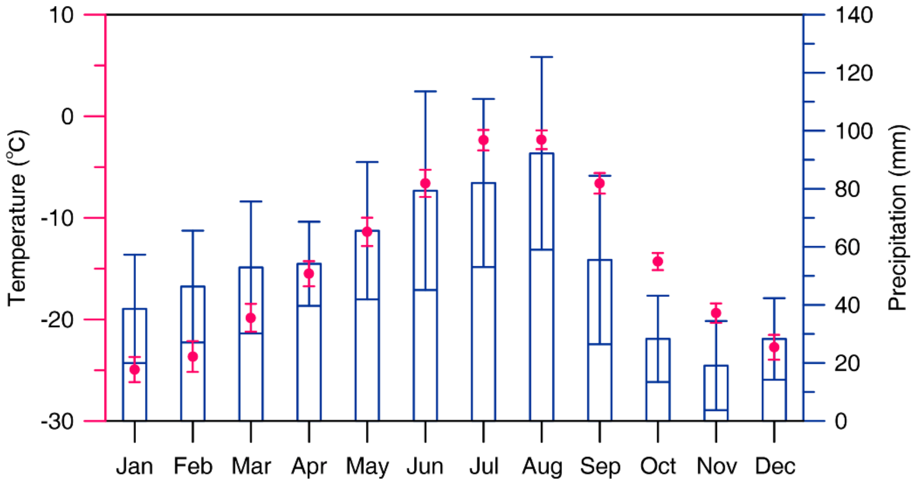

Fig. S5. Monthly mean temperature (red dots) and monthly precipitation (blue histogram) records in the vicinity of the Guliya ice cap during the period 1980-2022 CE. The temperature and precipitation data were extracted from the High Asia Refined analysis version 2 (*50*).

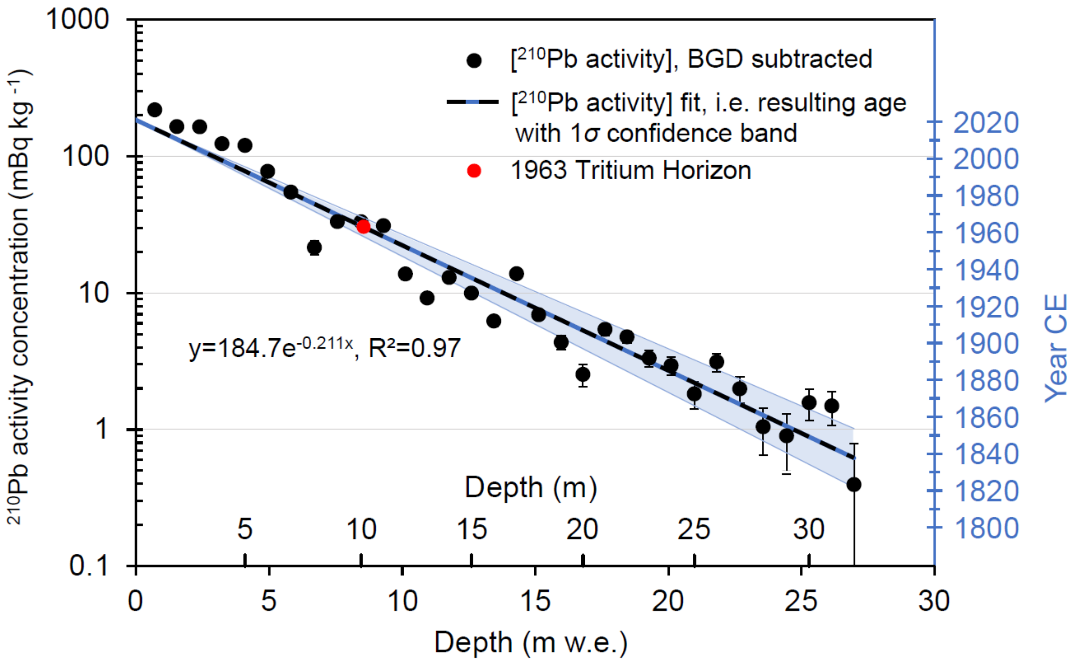

Fig. S6. ^{210}Pb activity concentration profile of the GP2021 ice core and the derived age–depth relationship. The ^{210}Pb background from supported lead (BGD; 1.5±0.3 mBq kg^{-1}) was subtracted. Note that the confidence band relates to the right-hand y-axis only. The time horizon from ^{3}H fallout associated with the year 1963 CE is in good agreement with the dating by ^{210}Pb. Thinning of annual layers from ice deformation was not considered, following standard procedure if the fit to the data does not significantly improve otherwise. However, the presence of moderate thinning cannot be ruled out as its observation might be masked by uncertainty of the data (scatter). Therefore, for the deep part, the lower (older) confidence limit may represent the most accurate age.

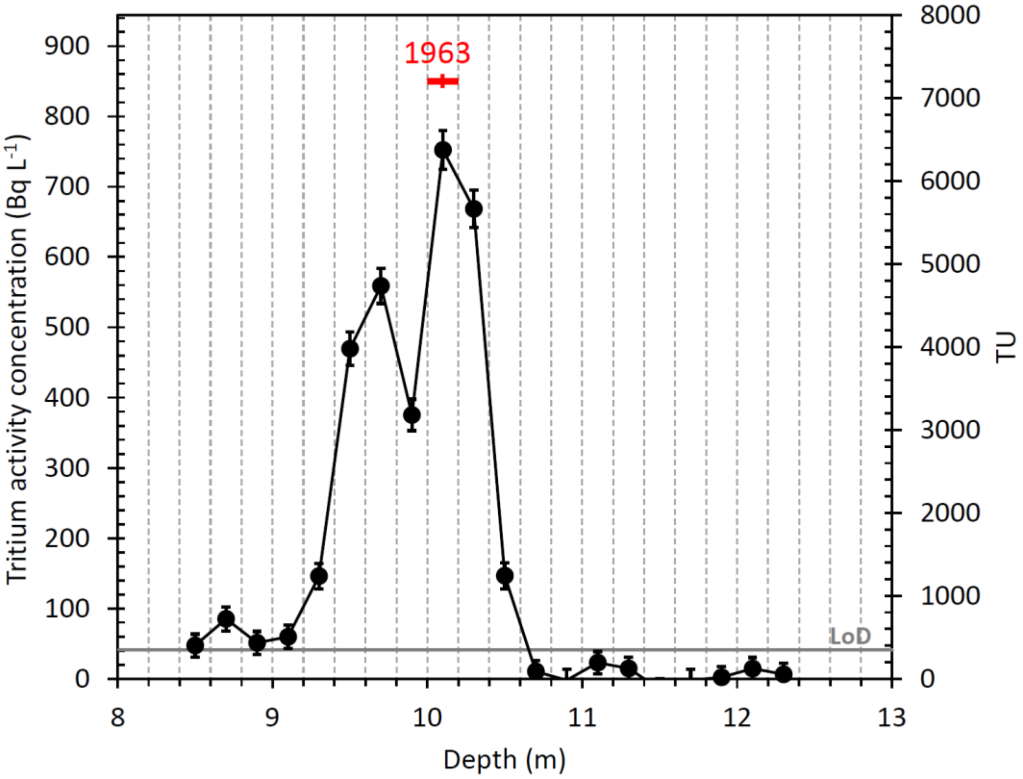

Fig. S7. Tritium activity concentration profile of the GP2021 ice core. Tritium activity concentrations are shown in units of Bq L^{-1} (left y-axis) and Tritium Units (TU, with 1 TU being 0.118 Bq L^{-1}; right y-axis). Displayed are the activity concentrations measured on 11 November 2021, corrected for the decay since 1963 CE (used as reference year for all samples). The analytical limit of detection (LoD) was 1.5 Bq L^{-1} (12.7 TU), for consistency, decay corrected for display here as well (grey line).

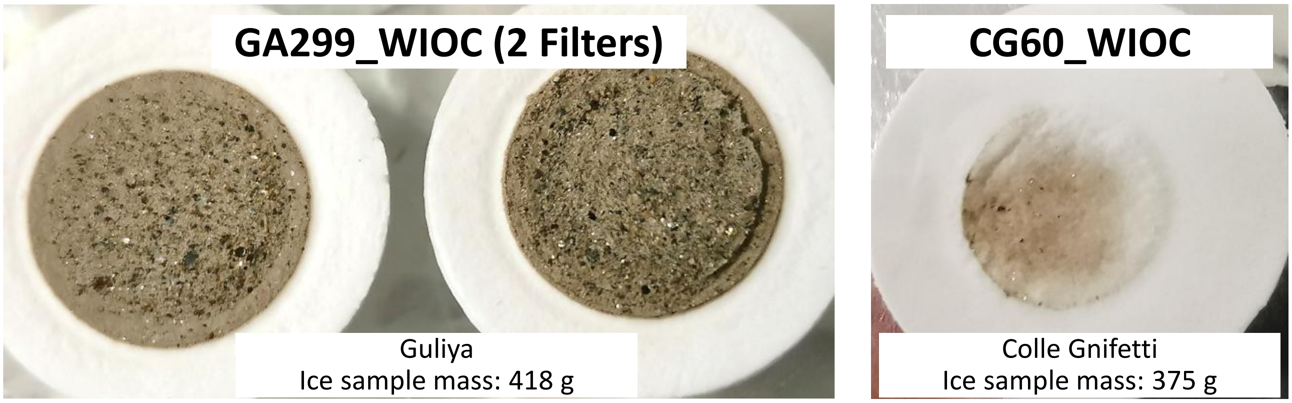

Fig. S8. Photos of filters after filtration of ice samples for the extraction of WIOC before subsequent ^{14}C analysis. Note the much higher mineral dust content in the sample from Guliya (loaded on two separate filters to prevent them from clogging) compared to a sample from a glacier in the European Alps (Colle Gnifetti).

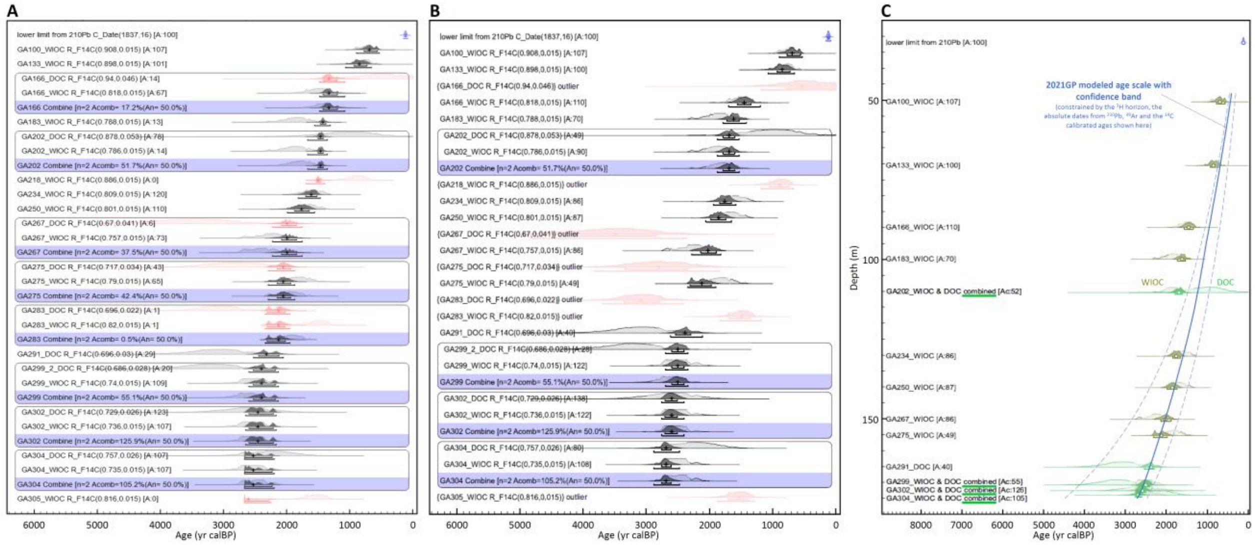

Fig. S9. ^{14}C calibration of the WIOC and DOC $F^{14}C$ results for the samples from the GP2021 ice core. Calibration was performed in OxCal v4.4.4 (*69*) with IntCal20 and post-bomb atmospheric curve of Northern Hemisphere zone 3 from (*67*), applying the in-built Bayesian sequence model (*71*, *72*). **(A)** Initial calibration run with enabling for calibration of combined WIOC and DOC results if available from the same depth interval. A priori age probability distribution functions (PDFs) from straight forward calibration are shown in light gray, posterior PDFs for sequential constraint in dark gray, and results of statistically identified outliers in red. **(B)** Same as in panel A but with identified outliers excluded for combination and sequence when re-running the calibration algorithm. **(C)** Final results as shown in panel B against depth, also showing the subsequently derived GP2021 timescale from combining all the independent ages from ^{210}Pb, ^{39}Ar and ^{14}C by the application of a one-dimensional ice flow model (blue line with dashed lines indicating its 1σ confidence envelope). See related text in the Supplement for more details. *Additional information:* The agreement index A provided in the text line for each sample indicates the level of agreement between the a priori and posterior PDFs. Values of 100 indicate no alteration in the distribution (100% agreement or unity) while values below 60 are considered critical solutions. Acomb denotes the agreement between combined results from the same layer. Combinations with Acomb < 50% also fail the chi-square test for statistically significant agreement at the 5% level. Results for which A was close to 0, or below 60 and at the same time failing the statistics for combination with the result from the other OC fraction analyzed in the same layer were considered as outliers (shown in red). Further details about the calibration algorithm and the statistics applied can be found in the OxCal online documentation (*69*), and the references therein.

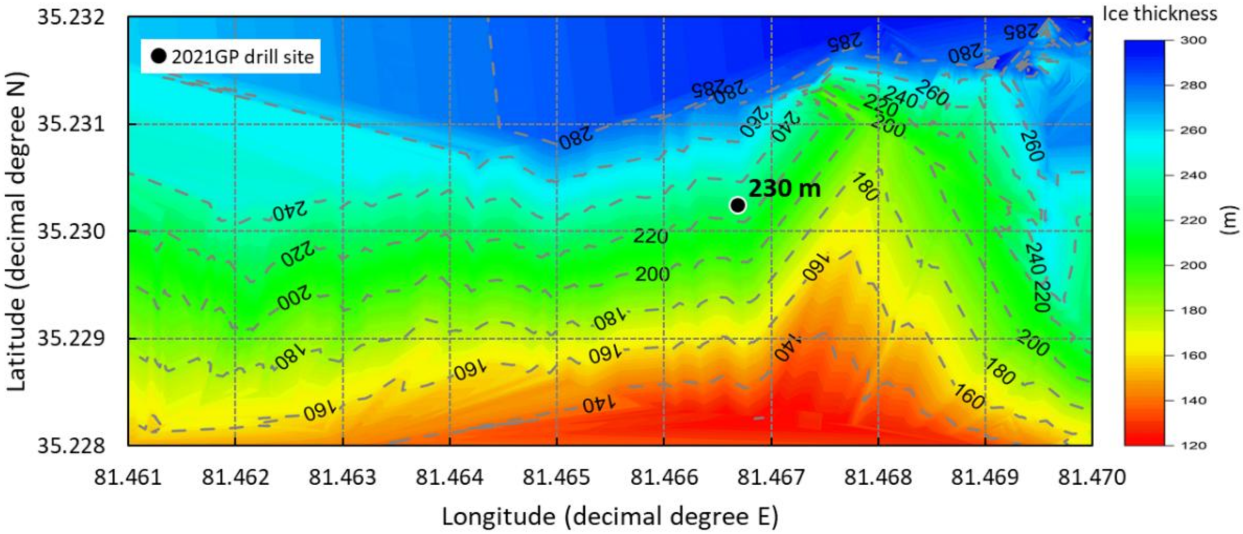

Fig. S10. Contour map of ice thickness based on ground penetrating radar measurements for the area covering the GP2021 ice core drill site. Data from ref. *74* deposited in the NOAA Paleo data repository. The closest GPR measurement was taken around 100 m to the North of the drill site, with tracks of measurements surrounding the site. From the contour map, an ice thickness at the site of around 230 m was estimated. The uncertainty is likely in the order of 30 to 40 m (based on the stated GPR measurement error of ±10-15 m, an estimated uncertainty of around 5 m related to a summed total of GPS position uncertainty of GPR data points and drill site, and around 10-20 m of uncertainty arising from contour mapping, i.e., the nudging of rather distant measurement points).

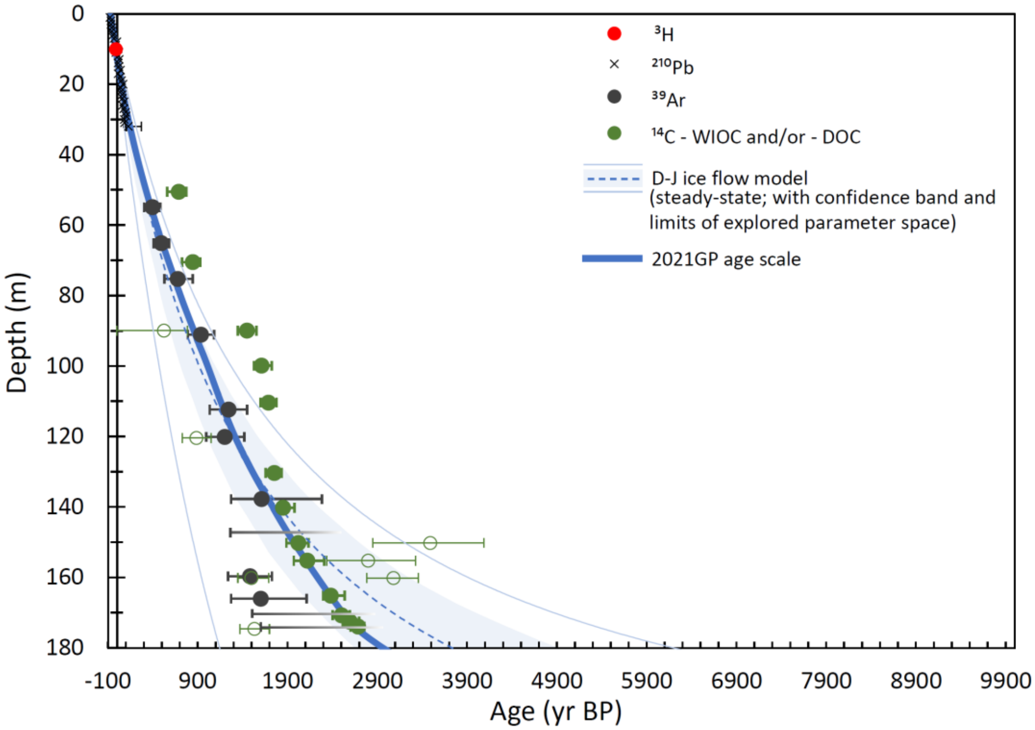

Fig. S11. The depth-age relationship of GP2021. The depth-age relationship of GP2021 (in blue) based on ice flow modeling results (Dansgaard-Johnsen model, i.e., D-J model; ref. *28*). They were constrained by the shown ages determined by absolute dating with ^{210}Pb, ^{39}Ar, ^{14}C and the ^{3}H horizon. The GP2021 age scale (thick blue line) was derived allowing for some variability in annual net accumulation rates (*b*; varying between 0.14 and 0.19 m w.e. yr^{-1}; *H*=250 m, *h*=*H*), while for the dashed line (*b*=0.16 m w.e. yr^{-1}; *H*=250 m, *h*=*H*) and the confidence band (blue shading; *b*=0.13, …, 0.18 m w.e . yr^{-1}; *H*=240, …, 270 m, *h*=*H*), values were kept constant; i.e. steady state was assumed. The thin, light blue lines indicate the lower (LL) and upper age limits (UL) derived from steady state ice flow modeling solutions for the full range of the explored parameter space (LL: *b*=0.21 m w.e. yr^{-1}, *H*=310 m, *h*=14; UL: *b*=0.12 m w.e. yr^{-1}, *H*=230 m, *h*=*H*; see Table S6).

^{14}C ages are either from the WIOC or DOC fraction, or if possible, represent a combination of both with open symbols indicating outliers based on the applied statistics for sequential deposition (see Fig. S9). For most ^{39}Ar samples below ~147 m depth, only a lower limit could be determined (horizontal black fading bars). This is not unexpected for samples with an age apparently at the limit of the dating range covered by this radionuclide (up to ~1800 years, ref. *23*).

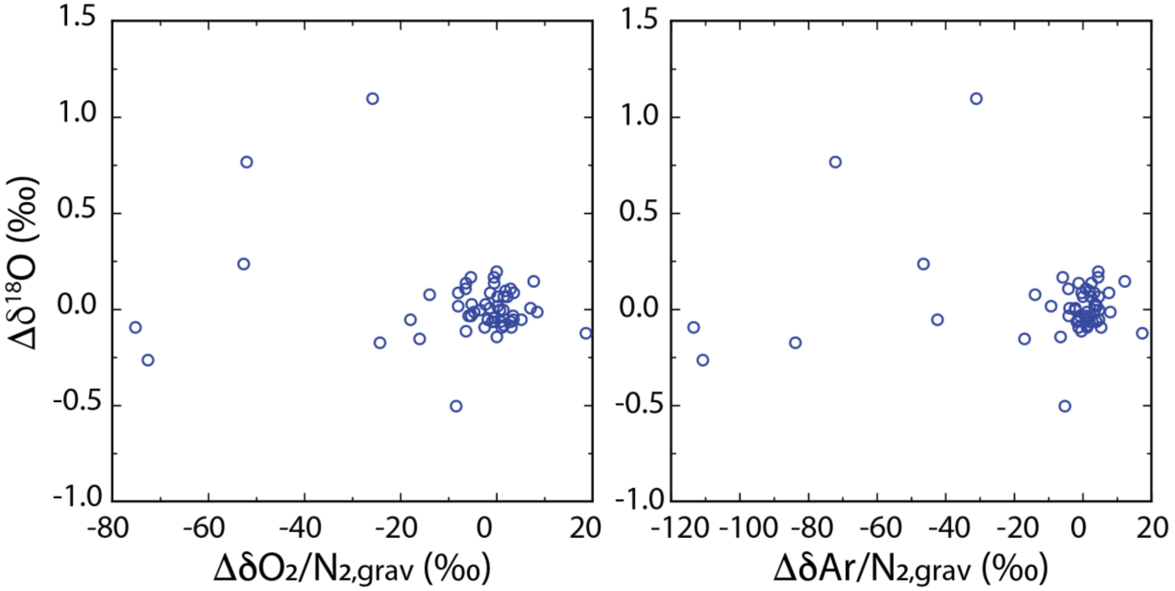

Fig. S12. Pair difference plot for $\Delta\delta^{18}O$-$\Delta\delta O_2/N_{2,grav}$ and $\Delta\delta^{18}O$-$\Delta\delta Ar/N_{2,grav}$. All data were corrected for gravitational fractionation. No obvious trend for gas loss can be observed.

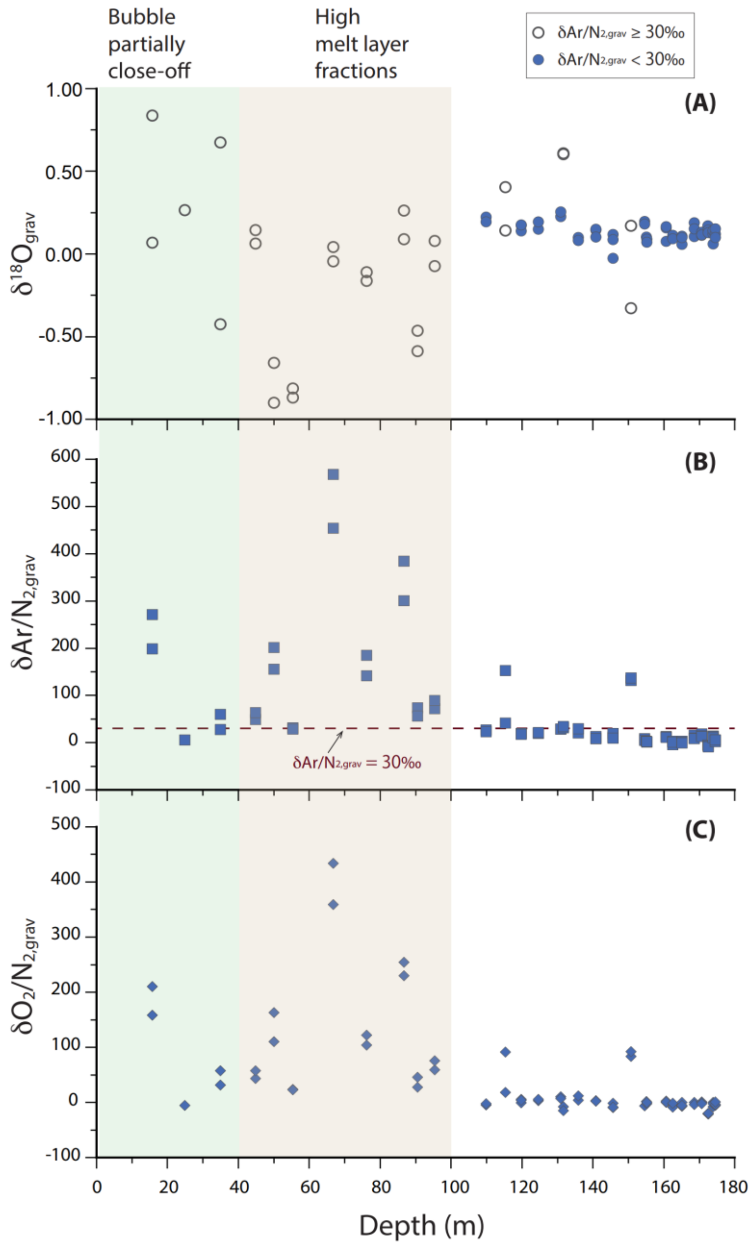

Fig. S13. Depth profiles of trapped gases. (**A**) $\delta^{18}O$; (**B**) $\delta Ar/N_{2,grav}$; (**C**) $\delta O_2/N_{2,grav}$. Filled circles in (A) represent samples with $\delta Ar/N_{2,grav}$ values less than 30‰. Open circles represent samples with $\delta Ar/N_{2,grav}$ values larger than 30‰, which are not considered for $\delta^{18}O_{atm}$ dating. The dashed line in (B) represents a $\delta Ar/N_{2,grav}$ ratio of 30‰.

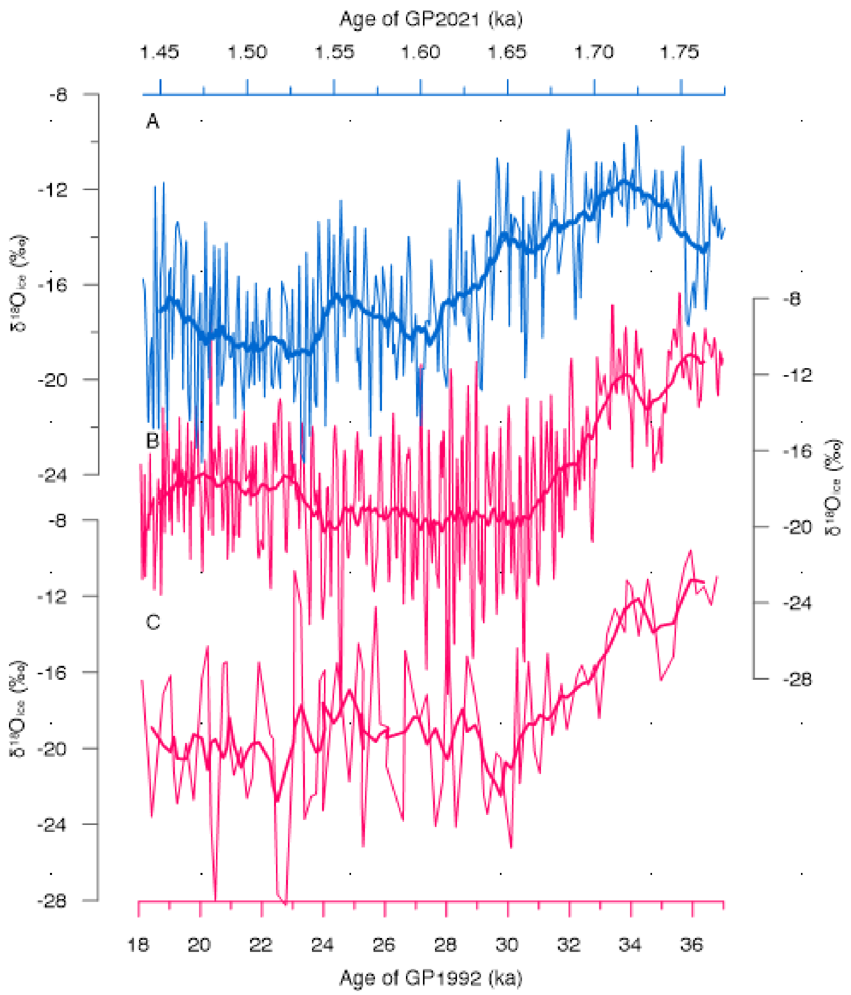

Fig. S14. The selected corresponding δ^{18}O$_{ice}$ profiles of GP2021 and GP1992 after their age matching as shown in Fig. 4A. (**A**) The annual-resolution δ^{18}O$_{ice}$ profile of GP2021 covering 1.44-1.77 ka (thin blue line). The thick blue line represents a 21-point running mean. (**B**) The 30-year-resolution δ^{18}O$_{ice}$ profile of GP1992 covering 18-37 ka (thin pink line) with the thick pink line representing a 41-point running mean. The GP1992 δ^{18}O$_{ice}$ data were extracted from ref. *84* using WebPlotDigitizer software. (**C**) The δ^{18}O$_{ice}$ profile of GP1992 covering 18-37 ka (thin pink line) with the thick pink line representing a 5-point running mean.

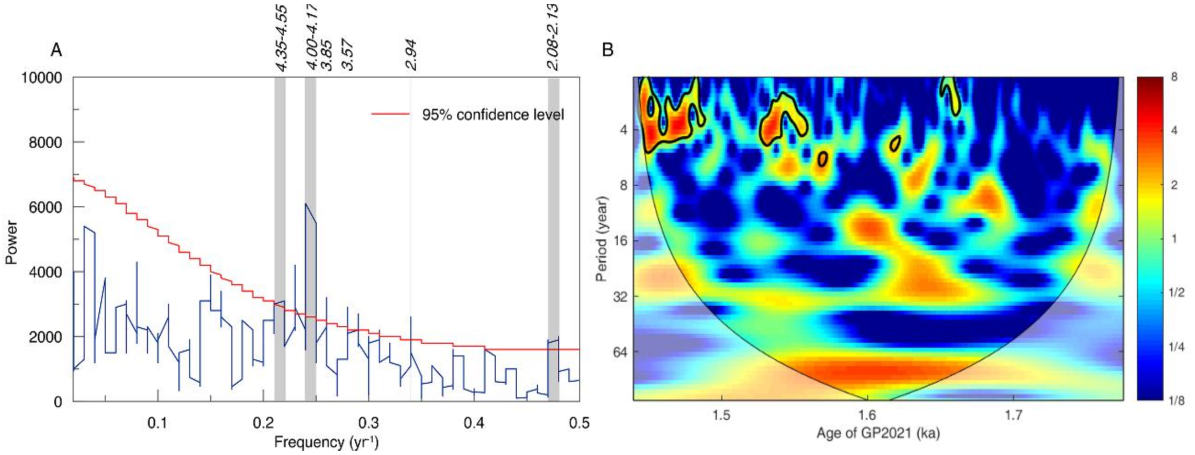

Fig. S15. Spectral analysis for the GP2021 $\delta^{18}O_{ice}$ record covering 1.44-1.77 ka. (**A**) Results from multitaper spectral analysis (*85*). (**B**) Results from Morlet wavelet transform spectra. The 95% confidence levels are presented as red line in frequency spectra (A) and black lines in Morlet wavelet transform spectra (B). Confidence levels are calculated relative to red-noise background signal (*89*, *90*). Gray bars mark periodicities of the GP2021 $\delta^{18}O_{ice}$ record covering 1.44-1.77 ka.

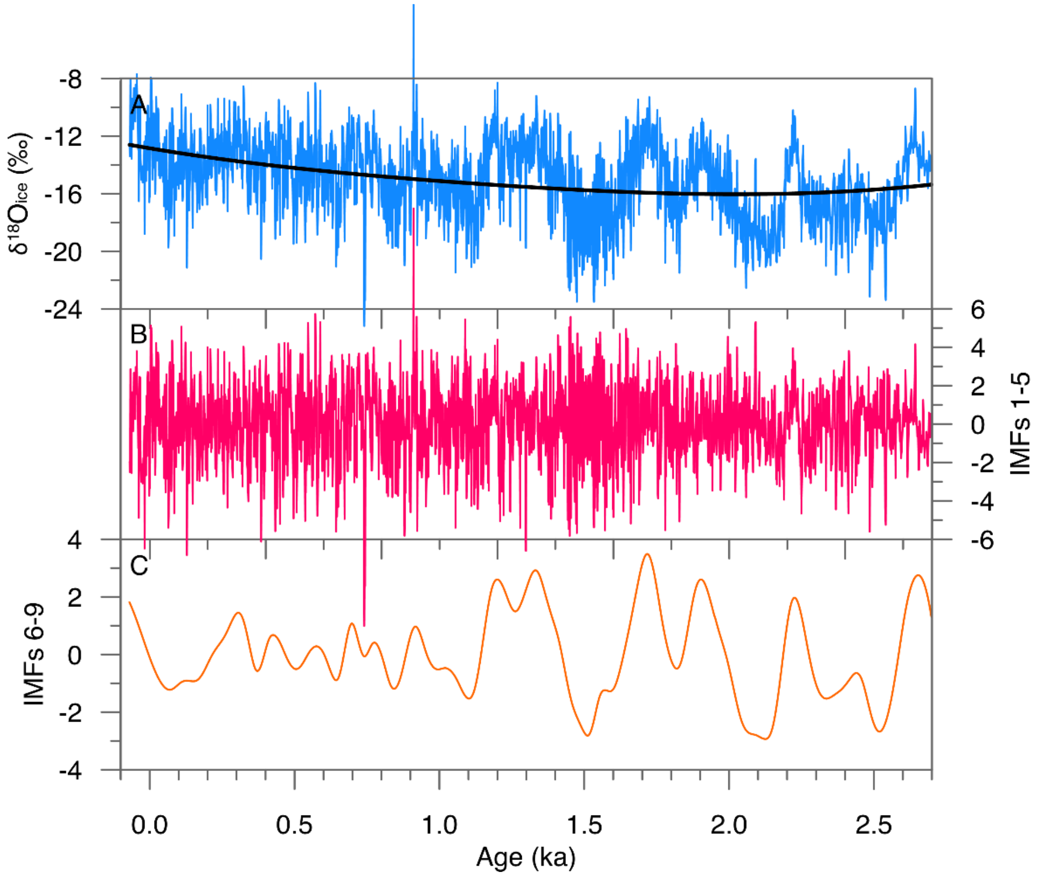

Fig. S16. The $\delta^{18}O_{ice}$ time series of the GP2021 ice core. (**A**) The black line indicates its long-term trend represented by IMFs 10-11 of the EEMD analysis (*91*). (**B**) The high-frequency fluctuations of the GP2021 $\delta^{18}O_{ice}$ represented by IMFs 1-5. (**C**) The GP2021 $\delta^{18}O_{ice}$ variations at multi-decadal to multi-centennial scales represented by IMFs 6-9.

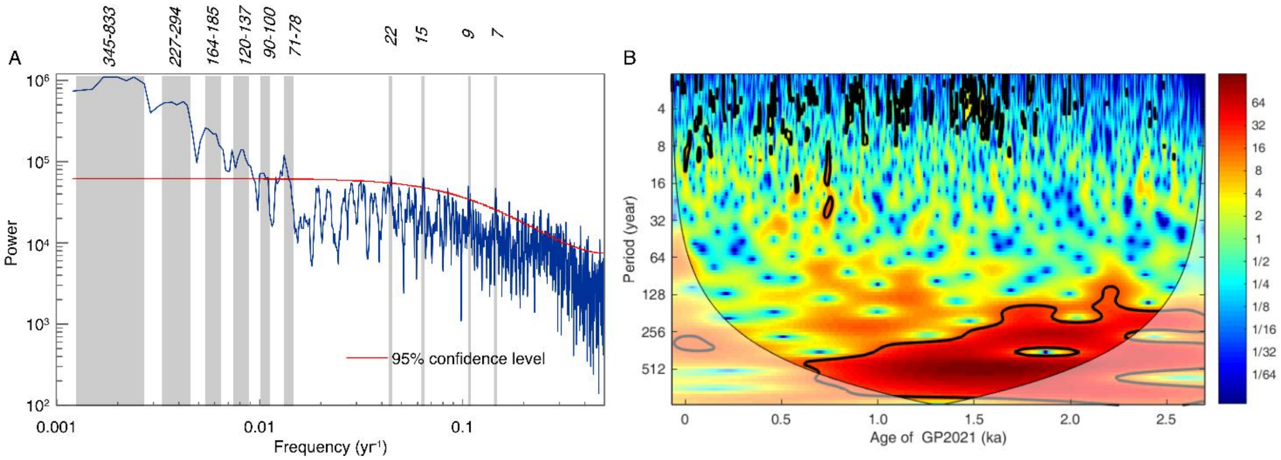

Fig. S17. Spectral analysis for the GP2021 $\delta^{18}O_{ice}$ record. (**A**) Results from multitaper spectral analysis (*85*). (**B**) Results from Morlet wavelet transform spectra. The 95% confidence levels are presented as red line in frequency spectra (A) and black lines in Morlet wavelet transform spectra (B). Confidence levels are calculated relative to red-noise background signal (*89*, *90*). Gray bars mark periodicities of the GP2021 $\delta^{18}O_{ice}$ record.

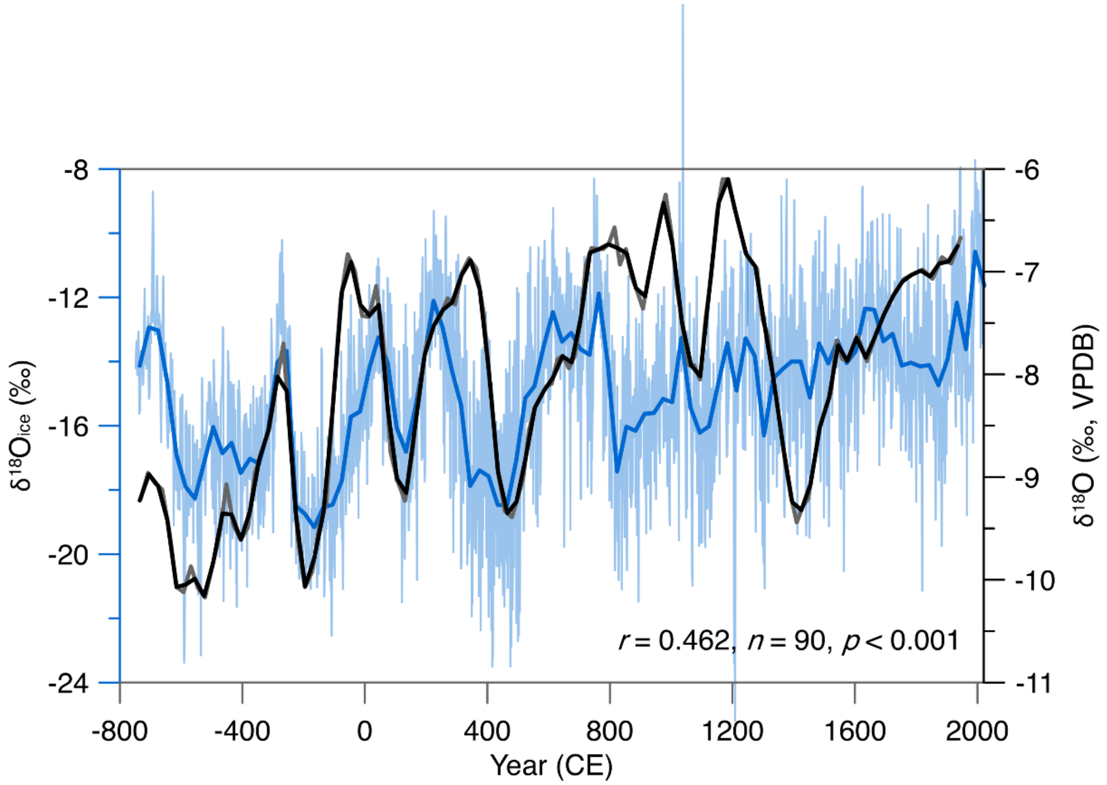

Fig. S18. The $\delta^{18}O_{ice}$ profile of GP2021 (blue lines) and the calcite $\delta^{18}O$ record of the Kesang stalagmites. The results of GP2021 and Kesang stalagmites based on their respective chronologies. The thin blue lines indicate the GP2021 annual means, and the thick blue lines indicate the GP2021 30-year means, while the thin grey lines indicate the Kesang raw data (*39*), and the thick black lines indicate the Kesang 30-year means**.**

Table. S1. The ^{36}Cl and Cl^- concentration data of GP1992.

Depth (m)	^{36}Cl (10^4 atoms g^{-1})	Cl^- (ppb)
10.420	0.210	472.877
13.880	0.149	461.978
40.000	0.213	526.100
62.590	0.313	537.324
82.360	0.242	463.573
136.070	0.271	559.596
170.710	0.289	842.949
178.450	0.326	808.142
179.500	0.234	808.317
180.500	0.291	784.517
181.500	0.329	736.750
182.550	0.323	686.595
183.600	0.452	673.860
184.650	0.234	689.190
185.650	0.295	703.790
186.650	0.319	662.035
204.550	0.246	575.535
218.760	0.220	635.024
223.500	0.155	607.100
239.530	0.211	586.579
249.970	0.231	541.648
254.170	0.217	491.672
263.630	0.240	446.401
286.140	0.179	674.720
291.370	0.186	578.433
296.550	0.117	446.640
305.440	0.054	96.816

Table S2. ^{210}Pb activity concentration [A] and resulting ages for the upper part of GP2021 (0.6-32.5 m depth). The ^{210}Pb background from supported lead (BGD) was 1.5±0.3 mBq kg^{-1}. The ages are additionally given in yr BP (before present with present = 1950 CE), which is used as input for ice flow modelling. Note that thinning of annual layers from ice deformation was not considered, following standard procedure if the fit to the data does not significantly improve otherwise. However, the presence of moderate thinning cannot be ruled out as it might be masked by analytical uncertainty.

Sample #	Depth (m)	Mid depth (m w.e.)	[A] BGD subtracted (mBq kg^{-1})	Year from fit and law of decay (CE)	Upper 1σ limit (CE)	Lower 1σ limit (CE)	Age (yr BP)
1	0.6 – 1.5	0.707	218.9 ± 6.3	2016.2	2016.6	2015.8	$-66.2^{+0.4}_{-0.4}$
2	1.5 – 2.5	1.537	165.1 ± 5.1	2010.6	2011.4	2009.6	$-60.6^{+0.9}_{-0.9}$
3	2.5 – 3.5	2.394	164.4 ± 5.3	2004.7	2006.1	2003.3	$-54.7^{+1.4}_{-1.4}$
4	3.5 – 4.5	3.232	123.7 ± 7.6	1999.0	2000.9	1997.1	$-49.0^{+1.9}_{-1.9}$
5	4.5 – 5.5	4.097	120.2 ± 4.7	1993.2	1995.5	1990.7	$-43.2^{+2.5}_{-2.4}$
6	5.5 – 6.5	4.946	77.6 ± 2.5	1987.4	1990.3	1984.4	$-37.4^{+3.0}_{-2.9}$
7	6.5 – 7.5	5.819	54.7 ± 2.2	1981.5	1984.8	1978	$-31.5^{+3.5}_{-3.4}$
8	7.5 – 8.5	6.701	21.5 ± 2.5	1975.5	1979.4	1971.4	$-25.5^{+4.0}_{-3.9}$
9	8.5 – 9.5	7.565	33.3 ± 1.3	1969.6	1974.0	1965.0	$-19.6^{+4.6}_{-4.4}$
10	9.5 – 10.5	8.458	33.2 ± 1.4	1963.5	1968.4	1958.4	$-13.5^{+5.1}_{-4.9}$
11	10.5 – 11.5	9.298	31.1 ± 0.5	1957.8	1963.2	1952.2	$-7.8^{+5.6}_{-5.4}$
12	11.5 – 12.5	10.120	13.8 ± 1.3	1952.2	1958.1	1946.1	$-2.2^{+6.1}_{-5.9}$
13	12.5 – 13.5	10.939	9.2 ± 0.6	1946.7	1953.0	1940.1	$3.3^{+6.6}_{-6.4}$
14	13.5 – 14.5	11.763	13.0 ± 0.7	1941.1	1947.9	1934.0	$8.9^{+7.1}_{-6.8}$
15	14.5 – 15.5	12.602	10.0 ± 0.6	1935.4	1942.7	1927.8	$14.6^{+7.6}_{-7.3}$
16	15.5 – 16.5	13.445	6.2 ± 0.5	1929.6	1937.5	1921.5	$20.4^{+8.1}_{-7.8}$
17	16.5 – 17.5	14.299	13.8 ± 1.2	1923.8	1932.1	1915.2	$26.2^{+8.6}_{-8.3}$
18	1.75 – 18.5	15.130	6.9 ± 0.6	1918.2	1927	1909.1	$31.8^{+9.1}_{-8.8}$
19	18.5 – 19.5	15.976	4.3 ± 0.5	1912.4	1921.7	1902.8	$37.6^{+9.6}_{-9.3}$
20	19.5 – 20.5	16.791	2.5 ± 0.5	1906.9	1916.7	1896.8	$43.1^{+10.1}_{-9.8}$
21	20.5 – 21.5	17.620	5.4 ± 0.5	1901.3	1911.5	1890.6	$48.7^{+10.6}_{-10.3}$
22	21.5 – 22.5	18.446	4.8 ± 0.5	1895.6	1906.4	1884.5	$54.4^{+11.1}_{-10.7}$
23	22.5 – 23.5	19.278	3.3 ± 0.5	1890.0	1901.2	1878.4	$60.0^{+11.6}_{-11.2}$
24	23.5 – 24.5	20.103	2.9 ± 0.5	1884.4	1896.1	1872.3	$66.6^{+12.1}_{-11.7}$
25	24.5 – 25.5	20.977	1.8 ± 0.4	1878.4	1890.6	1865.8	$71.6^{+12.6}_{-12.2}$
26	25.5 – 26.5	21.817	3.1 ± 0.5	1872.7	1885.4	1859.6	$77.3^{+13.1}_{-12.7}$
27	26.5 – 27.5	22.683	2.0 ± 0.4	1866.8	1880.0	1853.2	$83.2^{+13.6}_{-13.2}$
28	27.5 – 28.5	23.563	1.0 ± 0.4	1860.9	1874.6	1846.7	$89.1^{+14.2}_{-13.7}$
29	28.5 – 29.5	24.444	0.9 ± 0.4	1854.9	1869.1	1840.2	$95.1^{+14.7}_{-14.2}$
30	29.5 – 30.5	25.290	1.6 ± 0.4	1849.1	1863.8	1833.9	$100.9^{+15.2}_{-14.7}$
31	30.5 – 31.5	26.138	1.5 ± 0.4	1843.4	1858.6	1827.6	$106.6^{+15.7}_{-15.2}$
32	31.5 – 32.5	26.972	0.4 ± 0.4	1837.7	1853.4	1821.5	$112.3^{+16.2}_{-15.7}$

Table S3. The ^{39}Ar and ^{85}Kr data for the 2021 Guliya ice core samples. The given uncertainties are 1σ SDs, whereas upper limits are given at a 90% confidence level. The ^{85}Kr abundances below 50 m, originating from contamination, are not converted to age. The raw ^{39}Ar abundances as well as the ^{39}Ar abundances corrected for modern air contamination are provided. The shown ^{39}Ar ages are based on the corrected ^{39}Ar abundances.

Sample #	Depth (m)	Exact depth range (m)	Weight (kg)	^{85}Kr (dpm/cc)	^{39}Ar (pMAr)	^{39}Ar corr (pMAr)	^{39}Ar age (gas) (yr BP)
4	55	53.90 – 55.98	5.00	1.54 ± 0.16	$35.8^{+3.2}_{-2.9}$	$34.50^{+3.2}_{-3.0}$	365^{+35}_{-37}
5	65	64.21 – 66.09	4.92	0.37 ± 0.17	$26.5^{+2.5}_{-2.3}$	$26.20^{+2.5}_{-2.3}$	465^{+28}_{-31}
6	75	74.41 – 76.11	4.20	3.00	$18.6^{+2.3}_{-2.1}$	$15.40^{+4.1}_{-4.0}$	647^{+105}_{-87}
7	91	89.78 – 92.38	6.63	2.40 ± 0.22	$10.5^{+1.6}_{-1.4}$	$7.7^{+1.7}_{-1.5}$	905^{+76}_{-75}
9	113	111.46 – 113.28	6.81	1.65 ± 0.17	$5.5^{+1.4}_{-1.1}$	$3.5^{+1.4}_{-1.1}$	1210^{+130}_{-130}
10	120	118.98 – 121.35	6.22	0.97 ± 0.24	$5.1^{+1.5}_{-1.2}$	$3.9^{+1.5}_{-1.3}$	1170^{+140}_{-130}
13	138	136.75 – 138.84	5.40	2.45 ± 0.23	$4.3^{+1.1}_{-0.9}$	$1.3^{+1.2}_{-1.0}$	1580^{+580}_{-250}
15	147	146.23 – 148.32	5.31	0.70 ± 0.16	< 3.8	< 3.0	> 1260
17	160	158.74 – 160.65	4.95	0.43 ± 0.07	$2.3^{+1.0}_{-0.6}$	$1.8^{+1.0}_{-0.6}$	1450^{+160}_{-160}
18	166	165.02 – 167.05	4.94	1.17 ± 0.12	$2.7^{+1.1}_{-0.8}$	$1.3^{+1.1}_{-0.9}$	1570^{+420}_{-240}
19	170	169.20 – 171.31	5.29	0.67 ± 0.10	< 2.4	< 1.6	> 1500
20	174	173.07 – 175.10	4.92	0.87 ± 0.10	< 2.3	< 1.3	> 1590

The given uncertainty for the ^{39}Ar ages is based on the ^{39}Ar counting statistics as well as the error from the contamination correction. In addition, there is a systematic age uncertainty of 3% due to the error of the ^{39}Ar half-life, which would shift all ^{39}Ar ages up or down together. This error can be corrected and improved in the future with a more precise measurement of the ^{39}Ar half-life. Moreover, there is a systematic age uncertainty of about ±20 a due to the uncertainty of the atmospheric ^{39}Ar history (61). corr, corrected; dpm/cc, decay per minute per cubic centimeter STP of krypton; pMAr, percentage of the modern (year 2018 CE*) ^{39}Ar level.*

Table S4. Samples and results of ^{14}C dating. ^{14}C analyses of WIOC and DOC extracted from the GP2021 ice core. Provided are 1σ uncertainties, or for calibrated ages the 1σ range, respectively. Note that BP stands for before present, with present indicating the year 1950 CE. (see supplementary excel file)

Table S5. Input data for ice flow modeling and derived GP2021 ages at corresponding depths. Lower and upper confidence limits are denoted as LL and UL, respectively. See also Fig. S11.

**Dating Method**	**Sample ID**	**Mid depth** (m)	**Age** (yr BP)	**-1σ** (yr BP)	**+1σ** (yr BP)	**GP2021 age** (yr BP)	**LL** (yr BP)	**UL** (yr BP)
$^{210}Pb^{*}$	6	6.00	-43	-44	-42	-39	-39	-28
$^{210}Pb^{*}$	7	7.00	-32	-33	-31	-33	-34	-21
$^{210}Pb^{*}$	8	8.00	-2	-5	2	-28	-29	-13
$^{210}Pb^{*}$	9	9.00	-16	-17	-15	-22	-23	-6
$^{210}Pb^{*}$	10	10.00	-16	-17	-14	-17	-18	2
^{3}H	Peak maximum	10.10	-13	-14	-12	-16	-17	3
$^{210}Pb^{*}$	11	11.00	-14	-14	-13	-10	-12	10
$^{210}Pb^{*}$	12	12.00	13	10	16	-4	-7	17
$^{210}Pb^{*}$	13	13.00	26	24	28	2	-1	25
$^{210}Pb^{*}$	14	14.00	15	13	17	8	5	33
$^{210}Pb^{*}$	15	15.00	23	21	25	15	10	41
$^{210}Pb^{*}$	16	16.00	38	36	41	21	16	49
$^{210}Pb^{*}$	17	17.00	13	10	15	28	22	57
$^{210}Pb^{*}$	18	18.00	35	32	38	35	28	65
$^{210}Pb^{*}$	19	19.00	50	46	54	42	34	73
$^{210}Pb^{*}$	20	20.00	67	62	74	49	40	82
$^{210}Pb^{*}$	21	21.00	43	40	46	56	46	90
$^{210}Pb^{*}$	22	22.00	47	44	50	64	52	98
$^{210}Pb^{*}$	23	23.00	58	54	63	71	58	107
$^{210}Pb^{*}$	24	24.00	62	58	68	78	64	116
$^{210}Pb^{*}$	25	25.00	78	71	86	86	70	124
$^{210}Pb^{*}$	26	26.00	60	56	66	94	76	133
$^{210}Pb^{*}$	27	27.00	75	69	83	102	82	142
$^{210}Pb^{*}$	28	28.00	96	86	111	110	89	151
$^{210}Pb^{*}$	29	29.00	101	89	119	118	95	160
$^{210}Pb^{*}$	30	30.00	83	75	92	126	101	169
$^{210}Pb^{*}$	31	31.00	84	76	95	135	108	178
$^{210}Pb^{*}$	31	32.00	128	105	272	144	114	187
^{14}C	GA100_WIOC	50.568	687	560	770	328	246	377
^{39}Ar	Ar-4	54.940	395	305	483	378	280	427
^{39}Ar	Ar-5	65.150	495	408	579	505	365	554
^{14}C	GA133_WIOC	70.538	846	725	930	578	414	628
^{39}Ar	Ar-6	75.260	677	529	843	642	459	696
^{14}C	GA166_WIOC	89.953	1448	1346	1555	856	614	935
^{39}Ar	Ar-7	91.080	935	791	1080	872	627	956
^{14}C	GA183_WIOC	99.910	1612	1529	1724	1009	734	1126
^{14}C	GA202_combined	110.385	1687	1600	1780	1160	876	1358
^{39}Ar	Ar-9	112.370	1242	1034	1450	1189	906	1407
^{39}Ar	Ar-10	120.165	1202	995	1419	1315	1028	1612
^{14}C	GA234_WIOC	130.345	1754	1655	1834	1517	1207	1924
^{39}Ar	Ar-13	137.795	1612	1272	2282	1683	1356	2192
^{14}C	GA250_WIOC	140.240	1851	1743	1977	1739	1409	2288
^{14}C	GA267_WIOC	150.205	2021	1887	2134	1974	1646	2737
^{14}C	GA275_WIOC	155.265	2119	1970	2308	2108	1782	3004
^{39}Ar	Ar-17	159.700	1482	1236	1728	2226	1911	3267
^{14}C	GA291_DOC	165.190	2383	2298	2537	2376	2087	3635
^{39}Ar	Ar-18	166.035	1602	1273	2111	2401	2115	3697
^{14}C	GA299_combined	170.783	2503	2401	2593	2539	2285	4070
^{14}C	GA302_combined	172.575	2596	2512	2699	2596	2354	4225
^{14}C	GA304_combined	173.975	2688	2602	2757	2648	2409	4351

** Age of the individual data point and not the fit at given depth. See Materials and Methods: ^{210}Pb dating.*

Table S6. Exploration of the ice flow model parameter space for the GP2021 ice core drill site. The one-dimensional ice flow model applied, the Dansgaard-Johnsen model (*28*), assumes steady state conditions for the three parameters it is built on: the ice thickness *H*, the shear zone thickness *h*, and the average annual net accumulation rate *b*. These parameters were either pre-set or optimized for a best fit to the specified data set (denoted as free). Multiple variations were explored with pre-set values based on glaciological information such as GPR based ice thickness estimates or by applying different combinations of the dated horizons from the different, independent dating methods applied to the GP2021 ice core. See Table S5 for input datasets used, and Fig. 2A and Fig. S11 for modeling results.

Dataset used for parameter optimization	***H***		***h***[#]		***b***	
	setting	(m)	setting	(m)	setting	(m w.e. yr^{-1})
^{210}Pb, ^{3}H, ^{39}Ar, ^{14}C	free	275	free	275	free	0.162
	pre-set	230*	free	230	free	0.185
	pre-set	309[§]	pre-set	185[&]	free	0.149
^{3}H, ^{39}Ar	≤310	310	free	310	free	0.150
	≤270*	270	free	270	free	0.168
	pre-set	230*	free	14	free	0.132
^{3}H, ^{39}Ar data-subset <121 m	≤310	251	free	251	free	0.146
	≤270*	251	free	251	free	0.146
	pre-set	230*	free	230	free	0.153
^{3}H, ^{14}C	≤310	310	free	310	free	0.120
	≤270*	270	free	270	free	0.154
	pre-set	230*	free	230	free	0.186
^{3}H, ^{14}C data subset >121 m	≤310	277	free	277	free	0.149
	≤270*	270	free	270	free	0.154
	pre-set	230*	free	230	free	0.205
Full range shown in Fig. S10		230-310		$14 \leq h \leq H$		0.120-0.205
Confidence envelope shown in Fig. S10		240-270[$]		$h = H$		0.134-0.179[£]
Dashed line shown in Fig. S10		250[**]	$h = H$	250	average	0.157
**GP2021 timescale** (non-steady state *b*)		**250**		**250**	variable *b*	**0.137-0.191**

*[#]The theoretically maximal range for h is 0<h≤ H (constrained accordingly in the "free" setting). *The GPR based thickness estimate for the GP2021 drill site is 230 m with an uncertainty of around 40 m (see Fig. S10). [§]The bedrock depth of the GP1992 ice core was 309.7 m and 308.6 m for GP2015. [&]Gabrielli et al. (76) found h≈0.6*H to be a good approximation for a high-altitude glacier in the European Alps, with their results suggesting h to be around the depth of the firn-ice transition, reached at ~1 m depth in GP2021, what would suggest h≈H for this site. [$]The GPR based ice thickness was estimated with 230 m, all solutions for "free" parameter setting yielded H >250 m (240 m = mid value of 230 m and 250 m; selected as lower confidence limit). The GPR based maximal ice thickness estimate is around 270 m (selected upper confidence limit). [£]1σ-range of found values (average +/- 1 standard deviation). [**]Value obtained by fit optimization (rounded from the 251 m obtained for the ^{3}H and ^{39}Ar above 120 m depth) and as well in agreement with the GPR ice thickness estimate range.*

Table S7. Isotopic and elemental ratios of trapped air for GP2021.

Sample ID[a]	Top depth (m)	Bottom depth (m)	$\delta^{15}N$ vs.air (‰)	$\delta^{18}O$ vs.air (‰)	$\delta O_2/N_2$ vs.air (‰)	$\delta Ar/N_2$ vs.air (‰)	$\delta^{18}O_{atm}$ (‰)	$\delta O_2/N_{2,grav}$ (‰)	$\delta Ar/N_{2,grav}$ (‰)
GARun33-1	15.7	15.75	-0.862	-0.886	154.9	188.5	0.838	158.4	198.9
GARun33-2	15.7	15.75	0.177	0.421	211.3	273.3	0.068	210.6	271.2
GARun52-1	24.915	24.965	-2.192	-4.118	-14.0	-21.0	0.266	-5.2	5.3
GARun73-2	34.85	34.9	-0.061	-0.547	57.1	58.8	-0.426	57.3	59.5
GARun90-1	44.765	44.815	-0.351	-0.558	42.1	45.0	0.144	43.5	49.2
GARun90-2	44.765	44.815	0.115	0.292	58.0	64.7	0.062	57.5	63.3
GY-99	49.99	50.04	0.261	-0.136	111.0	158.0	-0.658	109.9	154.9
GY-99-B	49.99	50.04	0.238	-0.424	163.7	204.4	-0.901	162.7	201.6
GARun107-1	55.26	55.31	-0.081	-1.032	23.4	30.5	-0.869	23.7	31.5
GARun107-2	55.26	55.31	-0.012	-0.838	22.9	28.8	-0.814	23.0	28.9
GARun127-1	66.68	66.73	0.323	0.602	359.8	458.1	-0.045	358.5	454.2
GARun127-2	66.68	66.73	0.415	0.873	435.5	572.9	0.042	433.9	567.9
GARun144-1	76.11	76.16	0.022	-0.117	103.7	142.1	-0.162	103.6	141.8
GARun144-2	76.11	76.16	0.089	0.068	122.0	185.4	-0.110	121.7	184.4
GARun160-1	86.63	86.68	0.217	0.524	230.4	302.7	0.090	229.5	300.1
GARun160-2	86.63	86.68	0.182	0.628	254.7	386.3	0.263	253.9	384.1
GY-167	90.46	90.51	0.025	-0.539	45.8	74.1	-0.588	45.7	73.8
GY-167-B	90.46	90.51	-0.002	-0.468	27.3	56.6	-0.464	27.3	56.7
GARun176-1	95.33	95.38	0.188	0.302	60.0	73.9	-0.073	59.3	71.6
GARun176-2	95.33	95.38	0.146	0.372	76.1	90.6	0.080	75.5	88.8
GARun201-1	109.84	109.89	0.157	0.537	-4.0	28.8	0.224	-4.7	26.9
GARun201-2	109.84	109.89	0.129	0.451	-1.7	25.0	0.193	-2.2	23.5
GARun210-1	115.27	115.32	0.175	0.493	19.0	43.8	0.143	18.3	41.7
GARun210-2	115.27	115.32	0.232	0.867	91.9	155.5	0.404	91.0	152.7
GY-217-B	119.71	119.735	-0.047	0.079	5.6	18.7	0.173	5.8	19.3
GY-217	119.71	119.76	-0.087	-0.033	-0.9	18.1	0.140	-0.6	19.1
GY-217-C	119.735	119.76	-0.097	-0.019	3.9	17.1	0.175	4.3	18.3
GARun225-1	124.54	124.59	0.126	0.399	3.7	21.2	0.148	3.2	19.7
GARun225-2	124.54	124.59	0.099	0.392	5.6	22.5	0.193	5.2	21.3
GY-235	130.92	130.97	-0.108	0.009	10.1	27.6	0.225	10.5	28.9
GY-235-B	130.92	130.97	-0.104	0.047	6.8	27.2	0.255	7.3	28.4
GARun235-1	131.53	131.58	-0.162	0.285	-8.3	29.4	0.608	-7.7	31.3
GARun235-2	131.53	131.58	0.063	0.728	-14.4	34.5	0.603	-14.6	33.8
GARun243-1	135.81	135.86	-0.097	-0.093	3.6	19.2	0.101	4.0	20.4
GARun243-2	135.81	135.86	-0.004	0.074	12.1	29.8	0.082	12.1	29.9
GY-251-B	140.83	140.855	-0.115	-0.085	1.6	6.8	0.145	2.1	8.2
GY-251	140.83	140.88	-0.119	-0.087	2.9	11.3	0.150	3.4	12.7
GY-251-C	140.855	140.88	-0.130	-0.158	1.6	6.9	0.102	2.1	8.5
GY-259-B	145.57	145.595	-0.051	0.017	-9.6	12.0	0.119	-9.4	12.6
GY-259	145.57	145.62	0.015	0.004	-1.3	19.8	-0.026	-1.4	19.6
GY-259-C	145.595	145.62	-0.118	-0.149	-8.9	7.8	0.087	-8.5	9.2

GY-268	150.68	150.73	0.286	0.245	84.5	135.0	-0.327	83.4	131.6
GY-268-B	150.68	150.73	0.091	0.353	92.3	138.1	0.170	92.0	137.0
GARun274-1	154.45	154.5	0.228	0.655	-5.6	11.1	0.198	-6.5	8.4
GARun274-2	154.45	154.5	0.098	0.376	-6.1	5.7	0.181	-6.5	4.6
GY-275-B	155.07	155.095	-0.039	0.024	1.4	3.0	0.103	1.6	3.5
GY-275	155.07	155.12	-0.031	0.028	-2.1	0.6	0.090	-2.0	0.9
GY-275-C	155.095	155.12	0.022	0.114	0.5	2.0	0.070	0.4	1.8
GY-284-B	160.65	160.675	-0.093	-0.030	0.1	11.8	0.157	0.5	13.0
GY-284	160.65	160.7	0.005	0.085	2.9	12.5	0.076	2.9	12.5
GY-284-C	160.675	160.7	-0.078	0.011	0.8	10.4	0.166	1.1	11.3
GY-287-B	162.46	162.485	-0.104	-0.111	-3.0	2.0	0.098	-2.6	3.3
GY-287	162.46	162.51	-0.026	0.061	-8.5	-4.0	0.113	-8.4	-3.7
GY-287-C	162.485	162.51	-0.070	-0.049	-1.9	0.4	0.091	-1.6	1.2
GY-291-B	165.02	165.045	-0.033	-0.009	-0.3	3.1	0.057	-0.2	3.5
GY-291	165.02	165.07	0.011	0.128	-6.8	1.7	0.106	-6.8	1.5
GY-291-C	165.045	165.07	-0.032	0.038	-5.3	-1.4	0.101	-5.2	-1.0
GY-296-B	168.57	168.595	-0.059	0.069	-0.5	14.6	0.188	-0.2	15.4
GY-296	168.57	168.62	-0.088	-0.070	-0.6	7.9	0.105	-0.2	9.0
GY-296-C	168.595	168.62	-0.086	-0.020	-3.9	7.3	0.152	-3.5	8.3
GY-299-B	170.61	170.635	-0.048	0.033	1.2	17.9	0.130	1.3	18.5
GY-299	170.61	170.66	-0.034	0.044	-0.4	13.8	0.113	-0.3	14.2
GY-299-C	170.635	170.66	0.004	0.123	-1.8	12.8	0.116	-1.8	12.7
GY-302-B	172.42	172.445	0.041	0.250	-20.0	-5.0	0.169	-20.2	-5.5
GY-302	172.42	172.47	0.025	0.195	-20.5	-7.0	0.144	-20.6	-7.3
GY-302-C	172.445	172.47	-0.037	0.054	-19.9	-9.3	0.128	-19.7	-8.9
GY-304-B	173.82	173.845	-0.027	0.066	0.4	14.0	0.120	0.5	14.3
GY-304	173.82	173.87	0.047	0.154	-7.8	13.5	0.060	-8.0	12.9
GY-304-C	173.845	173.87	-0.007	0.128	-2.4	10.3	0.142	-2.4	10.4
GY-305-B	174.45	174.475	-0.028	0.064	-5.0	1.7	0.120	-4.9	2.1
GY-305	174.45	174.5	-0.046	0.060	-4.5	5.0	0.151	-4.3	5.5
GY-305-C	174.475	174.5	-0.005	0.090	1.0	6.1	0.100	1.0	6.1

REFERENCES AND NOTES

1. L. G. Thompson, T. Yao, M. E. Davis, K. A. Henderson, E. Mosley-Thompson, P.-N. Lin, J. Beer, H.-A. Synal, J. Cole-Dai, J. F. Bolzan, Tropical climate instability: The last glacial cycle from a Qinghai-Tibetan ice core. *Science* **276**, 1821–1825 (1997).

2. J. Jouzel, V. Masson-Delmotte, O. Cattani, G. Dreyfus, S. Falourd, G. Hoffmann, B. Minster, J. Nouet, J. M. Barnola, J. Chappellaz, H. Fischer, J. C. Gallet, S. Johnsen, M. Leuenberger, L. Loulergue, D. Luethi, H. Oerter, F. Parrenin, G. Raisbeck, D. Raynaud, A. Schilt, J. Schwander, E. Selmo, R. Souchez, R. Spahni, B. Stauffer, J. P. Steffensen, B. Stenni, T. F. Stocker, J. L. Tison, M. Werner, E. W. Wolff, Orbital and millennial Antarctic climate variability over the past 800,000 years. *Science* **317**, 793–796 (2007).

3. E. J. Brook, T. Sowers, J. Orchardo, Rapid variations in atmospheric methane concentration during the past 110 000 years. *Science* **273**, 1087–1091 (1996).

4. L. G. Thompson, J. P. Severinghaus, T. Yao, M. E. Davis, E. Mosley-Thompson, E. Beaudon, M. R. Sierra-Hernández, S. E. Porter, Use of $\delta^{18}O_{atm}$ in dating a Tibetan ice core record of Holocene/Late Glacial climate. *Proc. Natl. Acad. Sci. U.S.A.* **119**, e2205545119 (2022).

5. A. M. Seltzer, C. Buizert, D. Baggenstos, E. J. Brook, J. Ahn, J. Yang, J. P. Severinghaus, Does $\delta^{18}O$ of O_2 record meridional shifts in tropical rainfall? *Clim. Past* **13**, 1323–1338 (2017).

6. L. G. Thompson, T.-D. Yao, M. E. Davis, E. Mosley-Thompson, H.-A. Synal, G. Wu, J. F. Bolzan, S. Kutuzov, E. Beaudon, M. R. Sierra-Hernández, J. Beer, Ice core evidence for an orbital-scale climate transition on the Northwest Tibetan Plateau. *Quat. Sci. Rev.* **324**, 108443 (2024).

7. L. G. Thompson, T. Yao, M. E. Davis, E. Mosley-Thompson, G. Wu, S. Porter, B. Xu, P. Lin, N. Wang, E. Beaudon, K. Duan, M. Sierra-Hernández, D. Kenny, Ice core records of climate variability on the Third Pole with emphasis on the Guliya ice cap, western Kunlun Mountains. *Quat. Sci. Rev.* **188**, 1–14 (2018).

8. L. G. Thompson, M. E. Davis, E. Mosley-Thompson, T. Sowers, K. A. Henderson, V. S. Zagorodnov, P.-N. Lin, V. N. Mikhalenko, R. K. Campen, J. F. Bolzan, J. Cole-Dai, B.

Francou, A 25,000-year tropical climate history from Bolivian ice cores. *Science* **282**, 1858–1864 (1998).

9. L. G. Thompson, T. Yao, E. Mosley-Thompson, M. E. Davis, K. A. Henderson, P.-N. Lin, A high-resolution millennial record of the south Asian monsoon from Himalayan ice cores. *Science* **289**, 1916–1919 (2000).

10. L. G. Thompson, E. Mosley-Thompson, H. Brecher, M. E. Davis, B. Leon, D. Les, P.-N. Lin, T. Mashiotta, K. Mountain, Abrupt tropical climate change: Past and present. *Proc. Natl. Acad. Sci. U.S.A.* **103**, 10536–10543 (2006).

11. J. Wang, W. Wang, B. Cao, H. Cui, X. Chen, J. Qiu, M. Lei, J. Liao, Millennial-scale glacier fluctuations on the southeastern Tibetan Plateau during MIS 2. *Earth Planet. Sci. Lett*. **601**, 117903 (2023).

12. Z. P. Zhong, O. Zablocki, Y.-F. Li, J. L. van Etten, E. Mosley-Thompson, V. I. Rich, L. G. Thompson, M. B. Sullivan, Glacier-preserved Tibetan Plateau viral community probably linked to warm–cold climate variations. *Nat. Geosci.* **17**, 912–919 (2024).

13. PAGES2k Consortium, A global multiproxy database for temperature reconstructions of the Common Era. *Sci Data* **4**, 170088 (2017).

14. R. J. Delmas, J. Beer, H.-A. Synal, R. Muscheler, J.-R. Petit, M. Pourchet, Bomb-test ^{36}Cl measurements in Vostok snow (Antarctica) and the use of ^{36}Cl as a dating tool for deep ice cores. *Tellus* **56**, 492–498 (2004).

15. U. Heikkil, J. Beer, J. Feichter, V. Alfimov, H.-A. Synal, U. Schotterer, A. Eichler, M. Schwikowski, L. Thompson, ^{36}Cl bomb peak: Comparison of modeled and measured data. *Atmos. Chem. Phys.* **9**, 4145–4156 (2009).

16. L. Tian, F. Ritterbusch, J. Q. Gu, S. M. Hu, W. Jiang, Z.-T. Lu, D. Wang, G. M. Yang, ^{81}Kr dating at the Guliya ice cap, Tibetan Plateau. *Geophys. Res. Lett.* **46**, 6636–6643 (2019).

17. S. Hou, T. M. Jenk, W. Zhang, C. Wang, S. Wu, Y. Wang, H. Pang, M. Schwikowski, Age ranges of the Tibetan ice cores with emphasis on the Chongce ice cores, western Kunlun Mountains. *Cryosphere* **12**, 2341–2348 (2018).

18. S. Hou, W. Zhang, H. Pang, S.-Y. Wu, T. M. Jenk, M. Schwikowski, Y. Wang, Apparent discrepancy of Tibetan ice core $\delta^{18}O$ records may be attributed to misinterpretation of chronology. *Cryosphere* **13**, 1743–1752 (2019).

19. S. Hou, W. Zhang, L. Fang, T. M. Jenk, S. Wu, H. Pang, M. Schwikowski, Brief communication: New evidence further constraining Tibetan ice core chronologies to the Holocene. *Cryosphere* **15**, 2109–2114 (2021).

20. S. Hou, On the chronology of Tibetan ice cores. *Sci. Bull.* **67**, 2139–2141 (2022).

21. H. Gäggeler, H. von Gunten, E. Rössler, H. Oeschger, U. Schotterer, ^{210}Pb-dating of cold Alpine firn/ice cores from Colle Gnifetti, Switzerland. *J. Glaciol.* **29**, 165–177 (1983).

22. C. Wang, S. Hou, H. Pang, Y. Liu, H. W. Gäggeler, L. Tobler, S. Szidat, E. Vogel, ^{210}Pb dating of the Miaoergou ice core from the eastern Tien Shan, China. *Ann. Glaciol.* **55**, 105–110 (2014).

23. F. Ritterbusch, L. Tian, A. M. Tong, J. Q. Gu, W. Jiang, Z.-T. Lu, L. Shao, M. X. Tang, G. M. Yang, M. J. Zhang, L. Zhao, A Tibetan ice core covering the past 1300 years radiometrically dated with ^{39}Ar. *Proc. Natl. Acad. Sci. U.S.A.* **119**, e2200835119 (2022).

24. T. M. Jenk, S. Szidat, M. Schwikowski, H. W. Gäggeler, L. Wacker, H.-A. Synal, M. Saurer, Microgram level radiocarbon (^{14}C) determination on carbonaceous particles in ice. *Nucl. Instrum. Methods Phys. Res. B* **259**, 518–525 (2007).

25. C. Uglietti, A. Zapf, T. M. Jenk, M. Sigl, S. Szidat, G. Salazar, M. Schwikowski, Radiocarbon dating of glacier ice: Overview, optimization, validation and potential. *Cryosphere* **10**, 3091–3105 (2016).

26. W. Jiang, W. Williams, K. Bailey, A. M. Davis, S. M. Hu, Z. T. Lu, T. P. O'Connor, R. Purtschert, N. C. Sturchio, Y. R. Sun, P. Mueller, ^{39}Ar detection at the 10^{-16} isotopic abundance level with atom trap trace analysis. *Phys. Rev. Lett.* **106**, 103001 (2011).

27. H. Hu, Q. Li, W. Zhang, S. Wu, J. Song, K. Liu, X. Zou, H. Pang, M. Yan, S. Hou, δ^{18}O of O_2 in a Tibetan ice core constrains its chronology to the Holocene. *Geophys. Res. Lett.* **49**, e2022GL098368 (2022).

28. W. Dansgaard, S. J. Johnsen, A flow model and a time scale for the ice core from Camp Century, Greenland. *J. Glaciol.* **8**, 215–223 (1969).

29. A. Landais, G. Dreyfus, E. Capron, K. Pol, M.-F. Loutre, D. Raynaud, V. Y. Lipenkov, L. Arnaud, V. Masson-Delmotte, D. Paillard, Towards orbital dating of the EPICA Dome C ice core using $\delta O_2/N_2$. *Clim. Past* **8**, 191–203 (2012).

30. L. E. Lisiecki, P. A. Lisiecki, Application of dynamic programming to the correlation of paleoclimate records. *Paleoceanography* **17**, 1049 (2002).

31. W. S. Broecker, J. P. Kennett, B. P. Flower, J. T. Teller, S. Trumbore, G. Bonani, W. Wolfli, Routing of meltwater from the Laurentide Ice Sheet during the Younger Dryas cold episode. *Nature* **341**, 318–321 (1989).

32. T. Yao, V. Masson-Delmotte, J. Gao, W. Yu, X. Yang, C. Risi, C. Sturm, M. Werner, H. Zhao, Y. He, W. Ren, L. Tian, C. Shi, S. Hou, A review of climatic controls on δ^{18}O in precipitation over the Tibetan Plateau: Observations and simulations. *Rev. Geophys.* **51**, 525–548 (2013).

33. Z. Liu, Y. Bao, L. G. Thompson, E. Mosley-Thompson, C. Tabor, G. J. Zhang, M. Yan, M. Lofverstrom, I. Montanez, J. Oster, Tropical mountain ice core δ^{18}O: A Goldilocks indicator for global temperature change. *Sci. Adv.* **9**, eadi6725 (2023).

34. U. Büntgen, V. S. Myglan, F. C. Ljungqvist, M. McCormick, N. D. Cosmo, M. Sigl, J. Jungclaus, S. Wagner, P. J. Krusic, J. Esper, J. O. Kaplan, M. A. C. de Vaan, J. Luterbacher, L. Wacker, W. Tegel, A. V. Kirdyanov, Cooling and societal change during the Late Antique Little Ice Age from 536 to around 660 AD. *Nat. Geosci.* **9**, 231–236 (2016).

35. S. Feng, X. Liu, X. Mao, Vegetation dynamics in arid central Asia over the past two millennia linked to NAO variability and solar forcing. *Quat. Sci. Rev.* **310**, 108134 (2023).

36. T. Yao, Abrupt climatic changes on the Tibetan Plateau during the Last Ice Age-comparative study of the Guliya ice core with the Greenland GRIP ice core. *Sci China Ser D Earth Sci* **42**, 358–368 (1999).

37. T. Yao, B. Xu, J. Pu, Climatic changes on orbital and sub-orbital time scale recorded by the Guliya ice core in Tibetan Plateau. *Sci China Ser D Earth Sci* **44**, 360–368 (2001).

38. PAGES 2k Consortium, Continental-scale temperature variability during the past two millennia. *Nat. Geosci.* **6**, 339–346 (2013).

39. H. Cheng, P. Z. Zhang, C. Spötl, R. L. Edwards, Y. J. Cai, D. Z. Zhang, W. C. Sang, M. Tan, Z. S. An, The climatic cyclicity in semiarid-arid central Asia over the past 500,000 years. *Geophys. Res. Lett.* **39**, L01705 (2012).

40. W. An, S. Hou, Q. Zhang, W. Zhang, S. Wu, H. Xu, H. Pang, Y. Wang, Y. Liu, Enhanced recent local moisture recycling on the northwestern Tibetan Plateau deduced from ice core deuterium excess records. *J. Geophys. Res. Atmos.* **122**, 12541–12556 (2017).

41. H. Pang, W. Zhang, S. Wu, T. M. Jenk, M. Schwikowski, S. Hou, Abrupt climate fluctuations in Tibet as imprints of multiple meltwater events during the early to mid-Holocene. *Sci. Bull.* **69**, 375–381 (2024).

42. F. H. Chen, Z. C. Yu, M. L. Yang, E. Ito, S. M. Wang, D. B. Madsen, X. Z. Huang, Y. Zhao, T. Sato, H. J. B. Birks, I. Boomer, J. H. Chen, C. B. An, B. Wünnemann, Holocene moisture evolution in arid central Asia and its out-of-phase relationship with Asian monsoon history. *Quat. Sci. Rev.* **27**, 351–364 (2008).

43. W. Park, M. Latif, Atlantic Meridional Overturning Circulation response to idealized external forcing. *Clim. Dyn.* **39**, 1709–1726 (2012).

44. R. Zhang, R. Sutton, G. Danabasoglu, Y. Kwon, R. Marsh, S. G. Yeager, D. E. Amrhein, C. M. Little, A review of the role of the Atlantic meridional overturning circulation in Atlantic multidecadal variability and associated climate impacts. *Rev. Geophy.* **57**, 316–375 (2019).

45. S. Yu, M. S. Pritchard, A strong role for the AMOC in partitioning global energy transport and shifting ITCZ position in response to latitudinally discrete solar forcing in CESM1.2. *J. Clim.* **32**, 2207–2226 (2019).

46. Z. S. An, S. M. Colman, W. J. Zhou, X. Q. Li, E. T. Brown, A. J. T. Jull, Y. J. Cai, Y. S. Huang, X. F. Lu, H. Chang, Y. G. Song, Y. B. Sun, H. Xu, W. G. Liu, Z. D. Jin, X. D. Liu, P. Cheng, Y. Liu, L. Ai, X. Z. Li, X. J. Liu, L. B. Yan, Z. G. Shi, X. L. Wang, F. Wu, X. K. Qiang, J. B. Dong, F. Y. Lu, X. W. Xu, Interplay between the Westerlies and Asian monsoon recorded in Lake Qinghai sediments since 32 ka. *Sci. Rep.* **2**, 1–7 (2012).

47. M. Bender, T. Sowers, J. M. Barnola, J. Chappellaz, Changes in the O_2/N_2 ratio of the atmosphere during recent decades reflected in the composition of air in the firn at Vostok Station, Antarctica. *Geophys. Res. Lett.* **21**, 189–192 (1994).

48. H. Craig, Y. Horibe, T. Sowers, Gravitational separation of gases and isotopes in polar ice caps. *Science* **242**, 1675–1678 (1988).

49. V. B. Aizen, E. M. Aizen, D. R. Joswiak, K. Fujita, N. Takeuchi, S. A. Nikitin, Climatic and atmospheric circulation pattern variability from ice-core isotope/geochemistry records (Altai, Tien Shan and Tibet). *Ann. Glaciol.* **43**, 49–60 (2006).

50. X. Wang, V. Tolksdorf, M. Otto, D. Scherer, WRF-based dynamical downscaling of ERA5 reanalysis data for High Mountain Asia: Towards a new version of the High Asia Refined analysis. *Int. J. Climatol.* **41**, 743–762 (2021).

51. Y. Tang, H. Pang, W. Zhang, Y. Li, S. Wu, S. Hou, Effects of changes in moisture source and the upstream rainout on stable isotopes in precipitation—A case study in Nanjing, eastern China. *Hydrol. Earth Syst. Sci.* **19**, 4293–4306 (2015).

52. X.-Z. Dong, F. Ritterbusch, Y.-Q. Chu, J.-Q. Gu, S.-M. Hu, W. Jiang, Z.-T. Lu, G.-M. Yang, L. Zhao, Dual separation of Krypton and Argon from environmental samples for radioisotope dating. *Anal. Chem.* **91**, 13576–13581 (2019).

53. W. Jiang, K. Bailey, Z.-T. Lu, P. Mueller, T. P. O'Connor, C.-F. Cheng, S. M. Hu, R. Purtschert, N. C. Sturchio, Y. R. Sun, W. D. Williams, G.-M. Yang, An atom counter for measuring ^{81}Kr and ^{85}Kr in environmental samples. *Geochim. Cosmochim. Acta* **91**, 1–6 (2012).

54. G. J. Feldman, R. D. Cousins, Unified approach to the classical statistical analysis of small signals. *Phys. Rev. D* **57**, 3873–3889 (1998).

55. J. Q. Gu, A. L. Tong, G. M. Yang, S. M. Hu, W. Jiang, Z.-T. Lu, R. Purtschert, F. Ritterbusch, Reconstruction of the atmospheric ^{39}Ar/Ar history. *Chem. Geol.* **583**, 120480 (2021).

56. A. L. Tong, J.-Q. Gu, G.-M. Yang, S.-M. Hu, W. Jiang, Z.-T. Lu, F. Ritterbusch, An atom trap system for ^{39}Ar dating with improved precision. *Rev. Sci. Instrum.* **92**, 063204 (2021).

57. L. Fang, T. M. Jenk, T. Singer, S. Hou, M. Schwikowski, Radiocarbon dating of alpine ice cores with the dissolved organic carbon (DOC) fraction. *Cryosphere* **15**, 1537–1550 (2021).

58. T. M. Jenk, S. Szidat, D. Bolius, M. Sigl, H. W. Gäggeler, L. Wacker, M. Ruff, C. Barbante, C. F. Boutron, M. Schwikowski, A novel radiocarbon dating technique applied to an ice core from the Alps indicating late Pleistocene ages. *J. Geophys. Res.* **114**, D14305 (2009).

59. M. Sigl, T. M. Jenk, T. Kellerhals, S. Szidat, H. W. Gäggeler, L. Wacker, H.-A. Synal, C. Boutron, C. Barbante, J. Gabrieli, M. Schwikowski, Instruments and methods towards radiocarbon dating of ice cores. *J. Glaciol.* **55**, 985–996 (2009).

60. D. Lal, K. Nishiizumi, J. Arnold, In situ cosmogenic ^{3}H, ^{14}C, and ^{10}Be for determining the net accumulation and ablation rates of ice sheets. *J. Geophys. Res. Solid Earth* **92**, 4947–4952 (1987).

61. D. Lal, A. Jull, On determining ice accumulation rates in the past 40,000 years using in situ cosmogenic ^{14}C. *Geophys. Res. Lett.* **17**, 1303–1306 (1990).

62. D. Lal, “Cosmogenic in situ radiocarbon on the Earth” in *Radiocarbon After Four Decades* (Springer, 1992), pp. 146–161.

63. L. G. Thompson, E. Moslei-Thompson, M. E. Davis, P. N. Lin, J. Dai, J. F. Bolzan, A 1000-year climate ice-core record from the Guliya ice cap, China: Its relationship to global climate variability. *Ann. Glaciol.* **21**, 175–181 (1995).

64. T. Yao, L. G. Thompson, D. Qin, L. Tian, K. Jiao, Z. Yang, C. Xie, Variations in temperature and precipitation in the past 2 000 a on the Xizang (Tibet) Plateau—Guliya ice core record. *Sci. China Ser. D* **39**, 425–433 (1996).

65. Y. Shi, T. Yao, B. Yang, Decadal climatic variations recorded in Guliya ice core and comparison with the historical documentary data from East China during the last 2000 years. *Sci. China Ser. D Earth Sci.* **42**, 91–100 (1999).

66. H. M. Hoffman, “Micro radiocarbon dating of the particulate organic carbon fraction in Alpine glacier ice: Method refinement, critical evaluation and dating applications,” thesis, Ruperto-Carola Univ. of Heidelberg (2016).

67. P. J. Reimer, W. E. Austin, E. Bard, A. Bayliss, P. G. Blackwell, C. B. Ramsey, M. Butzin, H. Cheng, R. L. Edwards, M. Friedrich, The IntCal20 Northern Hemisphere radiocarbon age calibration curve (0–55 cal kBP). *Radiocarbon* **62**, 725–757 (2020).

68. Q. Hua, J. C. Turnbull, G. M. Santos, A. Rakowski, S. Ancapichún, R. de Pol-Holz, S. Hammer, S. Lehman, I. Levin, J. Miller, J. Palmer, C. Turney, Atmospheric radiocarbon for the period 1950-2019. *Radiocarbon* **64**, 723–745 (2022).

69. C. B. Ramsey, OxCal 4.4.4 calibration program, 2021. https://c14.arch.ox.ac.uk/oxcal/OxCal.html.

70. C. B. Ramsey, Radiocarbon calibration and analysis of stratigraphy: The OxCal program. *Radiocarbon* **37**, 425–430 (1995).

71. C. B. Ramsey, Deposition models for chronological records. *Quat. Sci. Rev.* **27**, 42–60 (2008).

72. C. B. Ramsey, Bayesian analysis of radiocarbon dates. *Radiocarbon* **51**, 337–360 (2009).

73. M. Fahnestock, W. Abdalati, I. Joughin, J. Brozena, P. Gogineni, High geothermal heat flow, basal melt, and the origin of rapid ice flow in central Greenland. *Science* **294**, 2338–2342 (2001).

74. S. Kutuzov, L. G. Thompson, I. Lavrentiev, L. Tian, Ice thickness measurements of Guliya ice cap, western Kunlun Mountains (Tibetan Plateau), China. *J. Glaciol.* **64**, 977–989 (2018).

75. J. F. Nye, The flow of glaciers and ice-sheets as a problem in plasticity. *Proc. R. Soc. Lond. A Math. Phys. Sci.* **207**, 554–572 (1951).

76. P. Gabrielli, T. M. Jenk, M. Bertó, G. Dreossi, D. Festi, W. Kofler, M. Winstrup, K. Oeggl, M. Schwikowski, B. Stenni, C. Barbante, Multimillennial synchronization of low and polar latitude ice cores by matching a time constrained Alpine record with an accurate Arctic chronology. *Climate Past Discuss.* Preprint (2022). DOI: 10.5194/cp-2022-20.

77. N. Wang, X. Jiang, L. G. Thompson, M. E. Davis, Accumulation rates over the past 500 years recorded in ice cores from the northern and southern Tibetan Plateau, China. *Arc. Ant. Alp. Res.* **39**, 671–677 (2007).

78. G. Dreyfus, F. Parrenin, B. Lemieux-Dudon, G. Durand, V. Masson-Delmotte, J. Jouzel, J.-M. Barnola, L. Panno, R. Spahni, A. Tisserand, U. Siegenthaler, M. Leuenberger, Anomalous flow below 2,700 m in the EPICA Dome C ice core detected using $\delta^{18}O$ of atmospheric oxygen measurements. *Clim. Past* **3**, 341–353 (2007).

79. S. Emerson, P. D. Quay, C. Stump, D. Wilbur, R. Schudlich, Chemical tracers of productivity and respiration in the subtropical Pacific Ocean. *J. Geophys. Res. Oceans* **100**, 15873–15887 (1995).

80. C. Huber, U. Beyerle, M. Leuenberger, J. Schwander, R. Kipfer, R. Spahni, J. P. Severinghaus, K. Weiler, Evidence for molecular size-dependent gas fractionation in firn air derived from noble gases, oxygen, and nitrogen measurements. *Earth Planet. Sci. Lett.* **243**, 61–73 (2006).

81. T. Sowers, M. Bender, D. Raynaud, Elemental and isotopic composition of occluded O_2 and N_2 in polar ice. *J. Geophys. Res. Atmos.* **94**, 5137–5150 (1989).

82. M. Bender, Orbital tuning chronology for the Vostok climate record supported by trapped gas composition. *Earth Planet. Sci. Lett.* **204**, 275–289 (2002).

83. R. C. Hamme, S. Emerson, The solubility of neon, nitrogen and argon in distilled water and seawater. *Deep. Sea Res. 1 Oceanogr Res. Pap.* **51**, 1517–1528 (2004).

84. M. E. Davis, "Climatic interpretations of eolian dust records from low-latitude, high-altitude ice cores," thesis, The Ohio State Univ., Columbus, OH, USA (2002).

85. M. Mann, J. Lees, Robust estimation of background noise and signal detection in climatic time series. *Clim. Change* **33**, 409–445 (1996).

86. B. Raup, A. Racoviteanu, S. J. S. Khalsa, C. Helm, R. Armstrong, Y. Arnaud, The GLIMS geospatial glacier database: A new tool for studying glacier change. *Global Planet. Change* **56**, 101–110 (2007).

87. NOAA National Centers for Environmental Information, *ETOPO 2022 15 Arc-Second Global Relief Model* (NOAA National Centers for Environmental Information, 2023).

88. T. Yao, L. G. Thompson, Map of the Guliya ice cap in west Kunlun Mountains of China (in Chinese), Xi'an Cartographic Publishing House, Xi'an, China (1992).

89. A. Grinsted, J. C. Moore, S. Jevrejeva, Application of the cross wavelet transform and wavelet coherence to geophysical time series. *Nonlin. Processes. Geophys.* **11**, 561–566 (2004).

90. D. R. Erbs-Hansen, K. L. Knudsen, J. Olsen, H. Lykke-Andersen, J. A. Underbjerg, L. Sha, Paleoceanographical development off Sisimiut, West Greenland, during the mid- and late Holocene: A multiproxy study. *Mar. Micropaleontol.* **102**, 79–97 (2013).

91. Z. Wu, N. E. Huang, Ensemble empirical mode decomposition: A noise-assisted data analysis method. *Adv. Adapt. Data Anal.* **1**, 1–41 (2009).
